# High-throughput characterization of cortical microtubule arrays response to anisotropic tensile stress

**DOI:** 10.1186/s12915-023-01654-7

**Published:** 2023-07-10

**Authors:** Elsa Demes, Stéphane Verger

**Affiliations:** 1 Umeå Plant Science Centre (UPSC), Department of Forest Genetics and Plant Physiology, Swedish University of Agricultural Sciences, 901 83 Umeå, Sweden; 2 Umeå Plant Science Centre (UPSC), Department of Plant Physiology, Umeå University, 901 87 Umeå, Sweden

**Keywords:** Plants, Microtubules, Mechanical stress, Image analysis

## Abstract

**Background:**

Plants can perceive and respond to mechanical signals. For instance, cortical microtubule (CMT) arrays usually reorganize following the predicted maximal tensile stress orientation at the cell and tissue level. While research in the last few years has started to uncover some of the mechanisms mediating these responses, much remains to be discovered, including in most cases the actual nature of the mechanosensors. Such discovery is hampered by the absence of adequate quantification tools that allow the accurate and sensitive detection of phenotypes, along with high throughput and automated handling of large datasets that can be generated with recent imaging devices.

**Results:**

Here we describe an image processing workflow specifically designed to quantify CMT arrays response to tensile stress in time-lapse datasets following an ablation in the epidermis — a simple and robust method to change mechanical stress pattern. Our Fiji-based workflow puts together several plugins and algorithms under the form of user-friendly macros that automate the analysis process and remove user bias in the quantification. One of the key aspects is also the implementation of a simple geometry-based proxy to estimate stress patterns around the ablation site and compare it with the actual CMT arrays orientation. Testing our workflow on well-established reporter lines and mutants revealed subtle differences in the response over time, as well as the possibility to uncouple the anisotropic and orientational response.

**Conclusion:**

This new workflow opens the way to dissect with unprecedented detail the mechanisms controlling microtubule arrays re-organization, and potentially uncover the still largely elusive plant mechanosensors.

**Supplementary Information:**

The online version contains supplementary material available at 10.1186/s12915-023-01654-7.

## Background

In plants, cortical microtubule (CMT) arrays tend to reorganize following the predicted tensile stress patterns [1]. This was first theorized in the 1960s [2, 3], indirectly inferred from observations more than forty years ago (e.g., [4]), and later experimentally shown in stretched plant tissues [5]. The topic has then gained significant interest in the last 15 years through progress in micromechanics, imaging, and computational modeling [6, 7]. Yet, we still do not understand how this is mediated. More specifically, if and how plants perceive mechanical stress, its orientation, and how this is transduced to the CMT arrays to change their overall organization. A few studies have started to uncover some of the molecular players involved [8–12], but these appear to be general regulators of CMT dynamics rather than specific regulators of the directional response to tensile stress. On the other hand, two recent studies investigated the role of the receptor-like kinase FERONIA as a putative mechanosensor for this response, but surprisingly found opposite results [13, 14]. While for both studies some of the conclusions were based on ablation experiments, the actual experiments and analysis of the data were performed in different ways and are difficult to compare. Overall, most studies on the question so far have focused on looking at only two and rarely more than two timepoints after mechanical stimulation, which limits our understanding of the dynamic response of CMT arrays to mechanical signals. Furthermore, many studies only focus on the characterization of the change in CMT arrays anisotropy rather than the change in orientation of the CMT arrays. This is due to the difficulty in predicting tensile stress patterns with which to compare the orientation of the CMT arrays at the cell and tissue level. Overall, a simple, accurate, and reproducible approach to assess CMT arrays tensile stress response with a high sensitivity and throughput needs to be established to detect even subtle phenotypes.

The epidermal ablation assay has proven to be a simple and robust method to change tensile stress patterns and study the response over time [6]. The epidermis being under tension [15] ablating one or several cells at its surface, rapidly creates a new circumferential tensile stress pattern that globally follows the geometry of the ablation site [6, 16]. While the damage to the ablated cells can induce many other responses, the changes in anisotropy and more importantly orientation of the CMT arrays, have clearly been linked to the presence of the tensile stress [6]. This assay is remarkably simple and can be performed in almost any lab simply using a fine needle for the ablation and a confocal microscope able to perform 3D time-lapse imaging. However, a major limiting step remains the image processing and quantification. Here, we have put together a largely automated high-throughput image processing workflow (https://github.com/VergerLab/MT_Angle2Ablation_Workflow) [17] specifically designed to quantify CMT arrays response to tensile stress in 3D time-lapse datasets following an ablation in the epidermis and we demonstrate its capacity to detect even subtle phenotypes.

## Results

### High-throughput analysis of CMT arrays dynamics

**Fig. 1:**
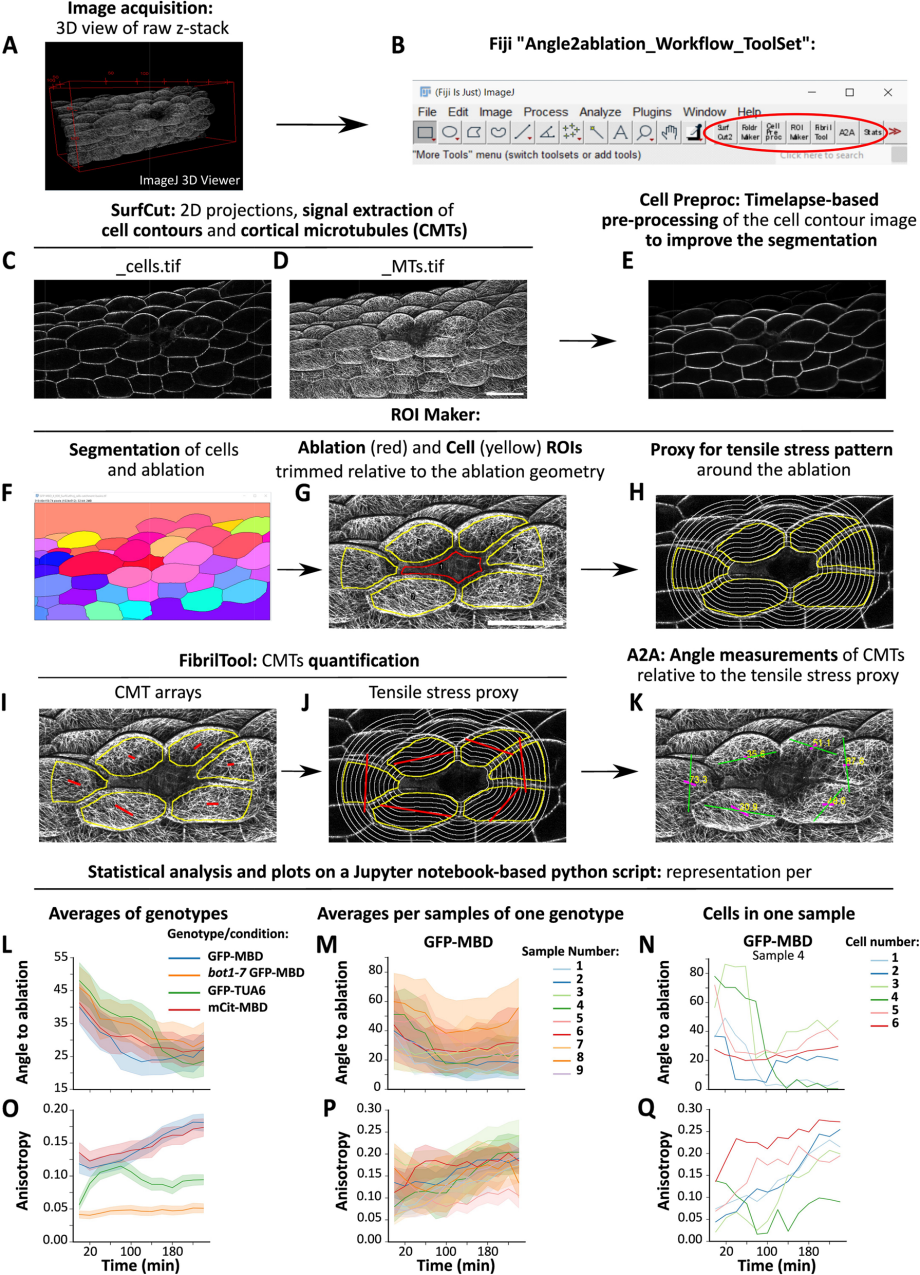
High-throughput image analysis workflow to quantify CMT arrays in hypocotyl cells. Panels describe each step of the image analysis strategy including the statistical analysis of the data. **A** 3D view of a raw confocal z-stack from an *Arabidopsis thaliana* light-grown hypocotyl expressing the GFP-MBD microtubule reporter line. **B** “Angle2Ablation_Workflow_Toolset” highlighted within the tool menu of ImageJ. **C**, **D** Epidermal cell contours (**C**) and CMTs outer epidermal signal (**D**) extracted from the raw z-stack and projected in 2D using the SurfCut ImageJ macro. **E** Average projection image of several cell contour images aligned from the same sample timelapse using the “Cell Preproc” tool. **F**–**H** Description of the analysis process of the “ROI Maker” macro. **F** MorphoLibJ morphological segmentation of the cell contour image. Note that while not all cells are perfectly segmented, those surrounding the ablation and the ablation site are correctly segmented. **G** 2D image of the outer epidermal CMTs overlayed with the ROIs of each cell region to analyze surrounding the ablation (yellow) and the ablation ROI (red). **H** Image of the geometry-based proxy for the tensile stress pattern, overlayed with the cell contour image and cell ROIs for context. **I**–**J** FibrilTool quantification of **I** the CMT arrays signal and **J** the tensile stress proxy. **K** Image of the outer epidermal CMT arrays overlayed with FibrilTool visual representation (lines) of anisotropy and angle for the CMTs (magenta) and the tensile stress proxy (green) as well as the angle difference (yellow) calculated with the “A2A” macro. **L**–**Q** Output of the statistical analysis done with a python script. Plots describing the angle to ablation and anisotropy for each genotype (**L**, **Q**), for each sample in a given genotype (**M**,** P**) or for each cell in a given sample (**N**,** Q**). In **L**,** M**,** O**, and **P**, the data plotted are the mean and the bootstrapped 95% confidence interval. Scale bars are 50 µm

The workflow takes as input 3D stacks from timelapse experiments with fluorescence signal from a microtubule reporter line (Fig. 1A). To streamline the use of the workflow, we have put together an ImageJ/Fiji toolset “Angle2ablation_Workflow_ToolSet.ijm” (Fig. 1B and Additional file 1) containing all the necessary macros, including those newly generated specifically for this analysis. The toolset can be easily installed by copying it in the “macros/toolset” folder of Fiji and then loading it from Fiji (see user guide for further details; Additional file 2). Each “tool” in the toolset runs a separate macro for a user-friendly experience and follows the steps of the workflow from left to right (Fig. 1B). The macro code remains also easily accessible and modifiable for more advanced and specific needs. In the first step, we use the pre-existing macro SurfCut2. As previously described in more detail [18, 19] SurfCut performs a binarization of the 3D confocal signal in order to detect the surface of the sample and uses it as a mask to crop specific layers of signal in the 3D volume following the surface of the sample, thus removing unwanted signal coming from the inside of the cell and underlying cells in the 3D stack. This allows the extraction and projection in 2D of the specific layers of signal coming from the outer epidermal CMTs on one hand (Fig. 1D), and of the cell contours of the epidermal cell layers on the other hand (Fig. 1C). The next tool in the toolset, “Foldr Maker,” is a simple script to generate a folder architecture (Experiment/Genotypes/Samples) to streamline the batch processing of the data with our workflow. The following tool, “Cell Pre proc,” is an optional preprocessing step before the cell segmentation. In this work, we have used samples expressing only a microtubule reporter, and no other way to clearly distinguish cell contours. While the cortical signal of the CMTs is often sufficient to automatically segment cell contours, the result is much more variable for the signal that is directly adjacent to the ablation. One solution is to use a reporter or dye that outlines the cell contours (e.g., plasma membrane or cell wall). Here, we instead make use of the temporal nature of the data to average the cell contour signal from each time point. In this “Cell Pre proc” macro, we use an affine registration algorithm to align each image of the timelapse to the first image, and then perform an average projection. For our dataset (4 h timelapse with 13 timepoints; one timepoint every 20 min) this step strongly improved the cell contour signal (Fig. 1E) and subsequent automatic segmentation (Fig. 1F). Note that depending on the situation (timelapse duration, interval during timepoints, sample growth rate, and cell divisions) the samples can sometimes considerably grow and change shape such that it may be difficult to register the images accurately throughout the whole timelapse. With this workflow, it is possible to define the number of images from the time series that should be registered and iteratively test the parameters that give the most useful result. With the next tool, “ROI Maker,” the image is first segmented into individual cells (including the ablation site) using the morphological segmentation tool from the ImageJ plugin MorphoLibJ (Fig. 1F) [20]. The macro allows subsequent manual correction of the segmentation if needed. The user is then prompted to select the ablation site within the segmented regions, from which the macro automatically selects a single row of cells directly adjacent to the ablation based on a region adjacency graph of the segmented labels. The selected cells and the ablation site are then eroded and converted into Regions of Interest (ROI) (Fig. 1G). The erosion step is needed to exclude the signal coming from the cell edges, for the subsequent CMT analysis. We then implemented within this macro a simple process to generate a proxy for the expected stress pattern around the ablation, to be used as a reference and compare it with the actual orientation of the CMT arrays (Fig. 1H). Based on previously reported computational simulations, the maximal tensile stress orientation is expected to generally follow circumferentially the geometry of the ablation [6, 16]. In following studies in which such simulations were not performed again, and based on this prior knowledge, the reference for the orientation of the maximal tensile stress was often estimated manually by drawing lines along the ablation site, which was thus highly prone to user bias [10, 13]. Here we reasoned that we could simply make use of the ablation geometry segmented in the previous step to generate this circumferential geometrical proxy in an unbiased manner. The segmented ablation shape is sequentially enlarged and used to draw white lines on a black background with a user-defined spacing and number of steps (Fig. 1H). Note that this approach is not an exact match for the actual expected stress pattern since tissue geometry-derived stress patterns and other pre-existing directional stress patterns (e.g., from differential growth) may already be present and could conflict to some extent with the new stress pattern generated by the ablation. It is also important to clarify that this is not a computation of tensile stress orientation but only a geometrical feature to assess the circumferential reorientation of the CMT arrays around the ablation site. Furthermore, it may only be adapted for tissues with relatively simple cell geometries. It may for instance not be adapted in the case of highly complex puzzle-shaped pavement cells, yet it can still give reasonable results in early stages of development (see Additional file 3: Supplemental Fig. S1). Alternatively, it is also possible to use the convex hull transformation of the ablation shape as the starting point for the generation of this proxy which can be more useful in certain situations (Additional file 3: Supplemental Fig. S1J and K). For our dataset, we expanded this geometrical proxy to 30 µm outward of the ablation corresponding to the average cell width in our samples. Furthermore, the macro uses this value to trim the previously defined ROI to only analyze the CMT signal coming from a fixed distance from the ablation site to limit some effects of the cell geometry in the quantification. Indeed, some elongated cells may have signal spanning far from the ablation site such that the CMTs in these regions may not be exposed to stress levels and patterns comparable with other cells which are fully enclosed within this defined perimeter (Fig. 1G, H and Additional file 3: Supplemental Fig. S2H and I). For the next tool, we have adapted the pre-existing macro FibrilTool [21, 22] to quantify the angles and anisotropy values in our datasets. In our workflow, FibrilTool can directly take as input the CMT images extracted with SurfCut (Fig. 1D) and the sets of ROIs generated after the automatic segmentation of the cells surrounding the ablation site (Fig. 1G). It operates in batch mode over the timelapse dataset and can be used to quantify the actual CMT arrays organization (Fig. 1I) as well as the geometrical proxy for tensile stress (Fig. 1J). Note that for those artificially generated images of tensile stress proxy, the anisotropy values measured by FibrilTool are irrelevant, but the angles measured provide the reference angles for the circumferential reorientation of CMTs. The next tool of the toolset, “A2A” (angle to ablation), is used to automatically perform the calculation of the angle difference between the circumferential proxy orientation and the actual CMT arrays orientation. A value of 0° would indicate perfect alignment, while 90°, an alignment perpendicular to the expected stress. The macro generates images overlaying the CMT signal and lines of FibrilTool visual output for both actual data and geometrical proxy as well as the angle difference values that were calculated (Fig. 1K). It also generates a single text file containing all the values for anisotropy and angle difference of each genotype/sample/cell/timepoints in a tidy format for further statistical analysis. Finally, we provide a Jupyter Notebook with a python-based data processing script for the plotting of data. Note that this notebook can be run online without any installation using the service “mybinder” (mybinder.org). For ease of use, the last tool in the toolset provides instructions and a link to the GitHub repository of the workflow where there is a link to this online version of the notebook. From there, the final text file generated by the A2A macro can be loaded and analyzed in a straightforward manner following instructions in the notebook. Interactive widgets included in the notebook provide a user-friendly way to display the data both under the form of tables and graphs at different levels (genotypes, individual samples, and individual cells, Fig. 1L–Q). This offers a full representation of the data to inspect a single data point as well as summary statistics (mean ± 95% confidence interval; see Additional file 4: Supplemental Table S1, S2, and S3 and Additional file 3: Supplemental Fig. S7, S8, S10, S11, S13, S14, S16, and S17). Concerning inferential statistics, here for simplicity's sake we use the bootstrapped 95% confidence (95CI) interval (as calculated by the seaborn python library) as an estimation of difference rather than a test of significance [23], since more advanced hypothesis testing would require complex case-by-case modeling of the curve's trends to be correct [24], which is out of the scope of this work but could be performed if needed on the data generated by the workflow.

Overall, we made an effort to guide all the steps of the process with a basic user interface/interaction within the Fiji macros and Jupyter notebook and have written a step-by-step user guide for the whole workflow. All described macros presented here work semi-automatically and in a batch mode. Log files are saved to follow and record the progress of the analysis. The steps in the workflow remove almost all the manual steps, virtually removing user bias or errors in the process and considerably shortening the analysis time.

Finally, the workflow is open and should be easy to modify with basic ImageJ macro knowledge. For instance, we use MorphoLibJ for segmentation and FibrilTool for CMT analysis, but these could be replaced by any plugin or process available in Fiji fitting the need of a specific analysis. In the future, we expect to generate derived versions of this workflow using the same overall framework, with the possibility to compare other structures (e.g., actin network and protein polarity) with other patterns (e.g., organ axis and cell aspect ratio).

### Workflow validation: CMTs array dynamic reorientation in response to tensile stress

To test our workflow, we chose to work with the commonly used microtubule reporter lines GFP-MBD (Microtubule Binding Domain) [25], the more recently designed mCit-MBD [26], and GFP-TUA6 (TUBULIN ALPHA-6) [27]. In the case of the GFP- and mCit-MBD lines, the fluorescent protein is fused to the microtubule-binding domain (MBD) of the mammalian Microtubule-associated protein 4 (MAP4) [25], which is generally believed to increase the stability of the CMT arrays and may preferentially bind or promote microtubule bundles [25]. Whereas, for GFP-TUA6, the fluorescent protein is fused to a tubulin subunit [27] which is believed to decrease CMT arrays stability. This line also displays a significant diffused cytoplasmic signal compared to the MBD reporter line [28]. In addition, we included the *botero1-7* GFP-MBD line [8], mutant for the microtubule severing enzyme katanin. It has been previously well characterized as a mutant impaired in its ability to reorganize its CMT arrays in response to changes in tensile stress [8, 16]. We performed ablations on 4-day-old light-grown hypocotyls and acquired 3D stacks every 20 min for 4 h (Additional file 3: Supplemental Fig. S3). We also performed mock experiments in which hypocotyls were not ablated but images were taken in the same conditions. We acquired timelapses for nine samples (biological replicates) for each genotype and each condition (with and without ablation) for a total of 72 timelapse, each containing 13 timepoints 3D stacks. All the microscopy data generated and analyzed for this study has been deposited at the Swedish National Data service (https://doi.org/10.5878/17te-jg54).

**Fig. 2:**
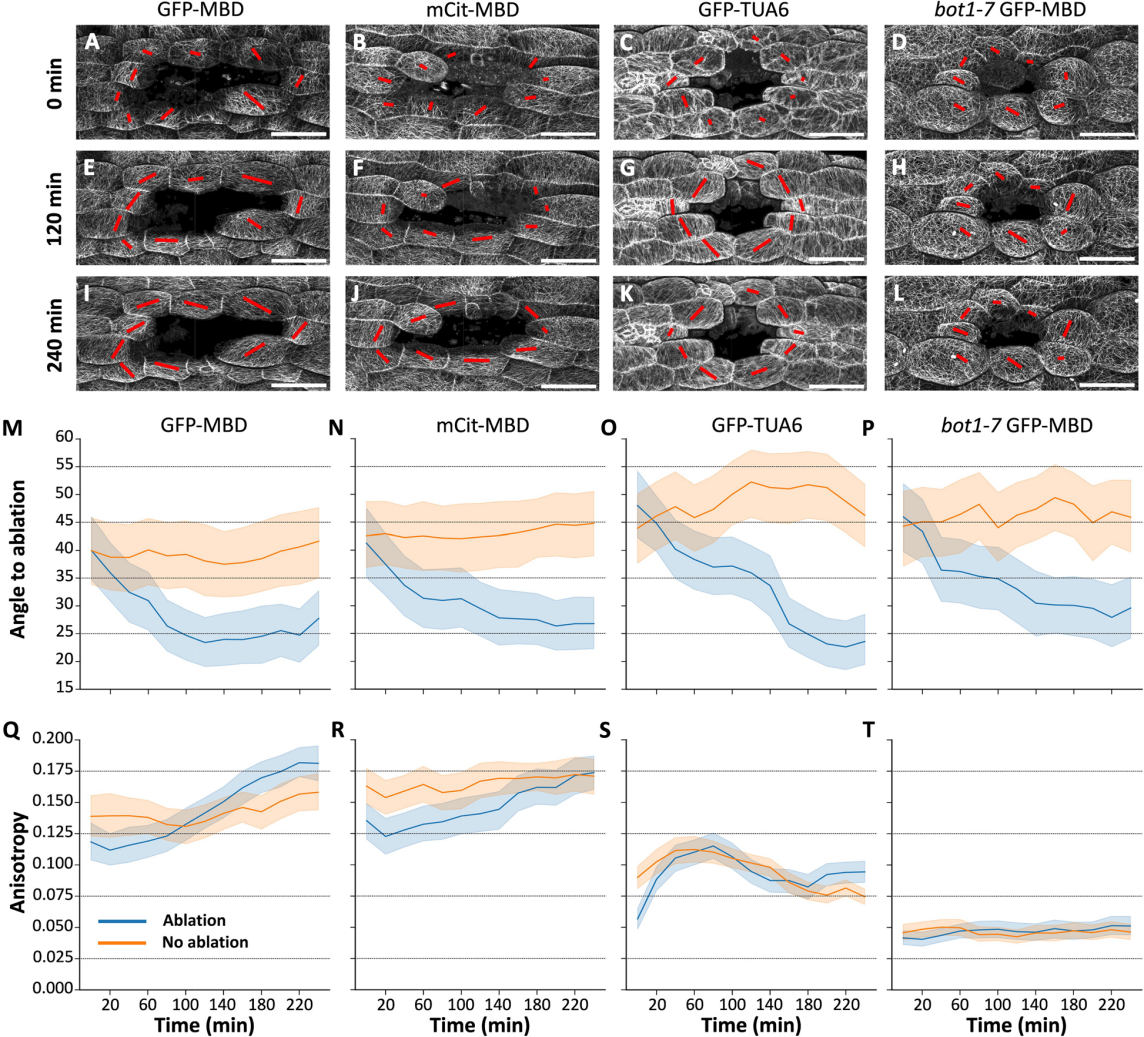
Timelapse quantification of CMTs reorganization on 4-day-old hypocotyls in response to tensile stress. **A**–**L** Confocal images of microtubule reporter lines, GFP-MBD (**A**, **E**, **I**), *bot1-7* GFP-MBD (**B**, **F**, **J**), mCit-MBD (**C**, **G**, **K**), and GFP-TUA6 (**D**, **H**, **L**) shown at three time points of the time series, 0, 120 and 240 min after the ablation (see full timelapse in Supplemental Fig. S6, S9, S12, and S15). The red lines represent the relative anisotropy by their length and the main orientation of the CMT arrays in the corresponding cells (scale bars are 50 µm). **M**–**T** Plots of the mean and bootstrapped 95% confidence interval for angle to ablation (**M**–**P**) and anisotropy (**Q**–**T**) for GFP-MBD (ablation: *n* = 81 cells in 9 hypocotyls; no ablation: *n* = 67 cells in 9 hypocotyls; **M** and **Q**), mCit-MBD (ablation: *n* = 75 cells in 9 hypocotyls; no ablation: *n* = 69 cells in 9 hypocotyls; **N** and** R**), GFP-TUA6 (ablation: *n* = 86 cells in 9 hypocotyls; no ablation: *n* = 73 cells in 9 hypocotyls; **O** and **S**) and *bot1-7* GFP-MBD (ablation: *n* = 71 cells in 9 hypocotyls; no ablation: *n* = 66 cells in 9 hypocotyls; **P** and **T**). The blue lines correspond to the samples with ablation and the orange lines, mock samples without ablation. The raw values used to generate this graph and the summary statistics plotted in the graphs can be found in Additional file 4: Supplemental Table S1, S2, and S3. All the data represented in this figure is also represented as average per samples as well as individual cells in Additional file 3: Supplemental Fig. S7, S8, S10, S11, S13, S14, S16, and S17

Based on a simple visual inspection of the dataset and as previously described, the CMT arrays of GFP-MBD, mCit-MBD, and GFP-TUA6 lines appeared to rearrange circumferentially around the ablation site over time while this is less obvious or visually appeared to be absent in the *bot1-7* GFP-MBD line (Fig. 2A–L). We have noted that for the GFP signal of GFP-TUA6 (Fig. 2C, G, K and Additional file 3: Supplemental Fig. S15), in some cells around the ablation site, the signal becomes so diffuse just after the ablation that it is impossible to identify individual CMTs. Those cells were not considered in the following analysis even though CMTs were often visible again after 20 min. This is likely due to the higher instability of CMT arrays in the GFP-TUA6 line, which may on the other hand be reduced in the MBD reporter lines. This unfortunately prevents the direct comparison of CMT arrays dynamics between these different reporter lines, at least when considering the anisotropy value quantified with FibrilTool.

Next, we analyzed this dataset with our new workflow. Based on visual observation and previous reports with quantifications on two timepoints [10, 13, 29], we expect a significant decrease in average angle values (circumferential reorientation) and an increase in anisotropy (increased alignment of CMT within a cell) over time for the reporter line. On the other hand, these values should remain stable in the absence of ablation. Following an ablation (blue lines), the quantified angle to ablation in the GFP-MBD reporter line drops from an initial value of 40 ± 6° (mean ± 95%CI), until it reaches a plateau starting at about 120 min with a value of 23 ± 4° (skewed towards a circumferential organization) and remains relatively stable until the end of the experiment (Fig. 2M). Similarly, the initial anisotropy value at *t*_0_ following the ablation (0.12 ± 0.01) increases over time to reach 0.18 ± 0.01 at *t*_240_ (Fig. 2Q). In the mock experiment (orange lines) we observed as expected that the angle and anisotropy remain stable with values ranging between 37 ± 4° and 41 ± 6° (lowest and highest angles) and 0.13 ± 0.01 and 0.16 ± 0.01 (lowest and highest anisotropies) throughout the time-series (Fig. 2M and Q). We observed the same overall behavior for the mCit-MBD reporter line (see Fig. 2N and R). Note that the average angle quantified at *t*_0_ or in the mock experiment is below the expected 45°, which reflects a bias due to a pre-existing longitudinal tensile stress in the hypocotyl along with the generally oval shape of the ablation in this tissue, as previously reported in [29].

To further validate the geometry-based proxy for circumferential reorganization, we compared the output of the workflow with results obtained when CMT angles were compared to manually drawn lines (Additional file 3: Supplemental Fig. S4). Those lines were placed as reported in previous studies [10, 13], by drawing a line between the two edges of the cell facing the ablation site. While this approach remains user biased (manual placement), it can also be considered to bring user “expertise” into the analysis. Ultimately, we see no significant difference in the output of the two approaches which suggest that our method is validated by “user expertise,” while it removes further bias from different users. Overall, our workflow works as expected and can recapitulate previous observations and quantitative reports. In turn, the timelapse-based nature of the analysis allows us to reveal the dynamic behaviors of CMT arrays, providing us with a clearer understanding of events taking place over time.

### Workflow’s detailed characterization reveals unexpected behaviors of CMT arrays in response to ablation

While the results generally appear to fit our original expectations, the detailed characterization provided by the high-resolution timelapse analysis in this workflow, reveals unexpected or previously unreported (to our knowledge) behaviors of the CMT arrays in response to the ablation.

First, in ablated GFP-MBD samples, the initial anisotropy value at *t*_0_ following the ablation (0.12 ± 0.01) is surprisingly lower and continues to drop at *t*_20_ (0.11 ± 0.02), compared with the mock sample (0.14 ± 0.02 and 0.14 ± 0.02) (Fig. 2Q). Similar results were observed for the mCit-MBD line. Due to experimental constraints, there is a 5- to 10-min delay between when the ablation is performed and when the first timepoint (“*t*_0_”) is acquired. We can assume that before the ablation, the anisotropy in the cells analyzed was equivalent to the non-ablated samples. Thus, the anisotropy value may have in fact dropped from about 0.14 ± 0.02 to 0.11 ± 0.02 in the first (approximately) 30 min following the ablation, before increasing again in the following timepoints. Furthermore, while there is a significant increase in anisotropy (non-overlapping 95CI) following an ablation between *t*_0_ to *t*_240_, the values throughout the timelapse consistently overlap in their 95CI with the non-ablated mock values. In turn, at *t*_240_ there appears to be only little (insignificant) difference in anisotropy between the ablated and non-ablated samples. When looking at these averaged values, this suggests that the ablation itself first induces a decrease in CMT anisotropy which is first recovered, rather than simply increasing from a non-perturbed initial anisotropy value. However, the effect is rather small, and it is important to consider the fact that this drop in anisotropy measured with FibrilTool may reflect a relative increase in cytoplasmic signal due to microtubules destabilization after the ablation (more obvious in the GFP-TUA6 line) rather than an actual disorganization of the CMT arrays.

Second, the GFP-TUA6 reporter line displays a response which when quantified with FibrilTool appears to some extent to be different in nature from what is observed with the MBD-based reporter lines, especially concerning the anisotropy. First, as expected, the angle to ablation does drop similarly to the MBD-based reporter lines, and the angles in the non-ablated samples, while showing some variation over time, do not drop (Fig. 2O). However, the anisotropy values show a surprising pattern. Both ablated and mock samples show a similar pattern starting with a low anisotropy which first increases fast and then decreases until reaching an apparent plateau (Fig. 2S). This suggests that, to a large extent, this pattern may be induced by the sample mounting and imaging. Nevertheless, similarly to the MBD-based reporter lines, when considering only the first and last time points, there is a significant increase in anisotropy after ablation, while in non-ablated samples the anisotropy value returns close to or even below the first observed value (Fig. 2S and Additional file 3: Supplemental Fig. S5). This suggest that the GFP-TUA6 line may in general be very sensitive to sample mounting and imaging condition, making it more difficult to study the actual effect of the response to the ablation with our workflow.

Finally, we included the katanin mutant line *bot1-7* GFP-MBD in which we expected to see a strongly impaired response for both angle and anisotropy values. As expected, there is a clear difference regarding overall anisotropy levels between the GFP-MBD and *bot1-7* GFP-MBD lines. For *bot1-7* GFP-MBD, it starts at and keeps a very low value throughout the experiment for both ablated and mock samples ranging from 0.04 ± 0.01 to 0.05 ± 0.01 (as compared to values increasing from 0.11 ± 0.02 to 0.18 ± 0.01 for GFP-MBD; Fig. 2T). However, contrary to the impression given by the visual observation, with this quantification we observed a clear drop in angle from 46 ± 6° at *t*_0_ to 30 ± 6° at *t*_240_, a response which appears to be indistinguishable from the GFP-MBD line alone (average angle dropped by about 15° in both cases; Fig. 2P). Note that while the low anisotropy values in the *bot1-7* mutant may limit our interpretation of the change in CMT arrays angles [21], our mock experiments confirm that this pattern is not random and does reveal a change of CMT arrays overall orientation that is not striking on the images but that can be measured with computer-assisted image analysis. This suggests that our workflow allows us to uncouple the orientation and anisotropic response which could open the door to genetically dissect the different components and molecular players involved in this response.

## Discussion

Here we describe a highly automated image processing workflow specifically designed to quantify CMT arrays response to tensile stress in time-lapse datasets following an ablation in the epidermis. With this workflow, we also introduce a new simple geometry-based method to generate a proxy for the circumferential stress pattern around the ablation. We were able to validate our new approach and workflow by reproducing expected quantitative observations of changes in CMT arrays angle relative to the ablation site as well as the increase in anisotropy following the ablation. In turn, this workflow enabled us to follow in fine detail the rearrangements of CMT arrays following a change in tensile stress pattern. This revealed, to our knowledge, some previously unreported behaviors. We could for instance observe that the ablation appears to induce a short-term drop of anisotropy (as measured by FibrilTool) that is then recovered. We could also reveal a very different behavior for the MBD-based and TUA-based reporter lines in our assay, which could notably explain some differences in conclusions reached in different studies using either of these reporter lines [13, 14]. Finally, we could also clearly uncouple the orientation and anisotropic response both through the characterization of the dynamic reorganization of CMTs in the reporter lines alone but also from the comparison with the response in the katanin mutant.

It is worth noting that in this study we only report observations and quantifications made on *Arabidopsis thaliana* light-grown hypocotyls, using reporter lines that influence the behavior of CMT arrays themselves. There may also be significant differences in the way CMT arrays respond to ablation in different tissues, other reporter lines, and species. The workflow is in principle applicable to a large variety of sample types but note that in some cases, (1) the cell and ablation shape complexity can limit the interpretability of the output, (2) additional signal acquisition from a cell wall or membrane marker may be required for the workflow to work efficiently, and (3) sample growth, deformation and the presence of cell divisions may limit the applicability of the workflow for long time-lapse or fast-growing and dividing samples. For instance, due to the puzzle-like shape of the pavement cells of the epidermal tissue of leaves and cotyledons, the resulting ablation shape could show large concave indentations that may limit the usefulness of our geometry-based proxy for tensile stress. It is not clear yet how the stress would propagate at the cellular and supracellular levels in lobes of pavements cells protruding towards the ablation. Yet, as shown in Additional file 3: Supplemental Fig. S1, when pavement cells show a rather low complexity as is often the case in young organs, the workflow appears to be applicable as is. Similarly for tissues with highly elongated cells, due to the pronounced oval geometry of the resulting ablation and likely strong impact of pre-existing differential growth-derived stress patterns [29], it is unclear whether our geometrical proxy for stress pattern would be useful. On the other hand, the shoot apical meristem, with its small and often isodiametric cells may appear to represent an ideal tissue for the analysis with our workflow. In this case, however, the CMT array signal is often very anisotropic, such that it is generally impossible to use the microtubule signal for the cell contour image generation. But this is not a major limitation since it is quite straightforward to use a cell wall or plasma membrane marker and record a second channel, which can then be processed easily with our workflow. The shoot apical meristem may also display a significantly higher cell division rate, which may require the exclusion of divided cells from the analysis as our workflow is not able to process such case. Thus, while most of these limitations can be largely alleviated, we found that the light-grown hypocotyls represented a more practical tissue for this analysis. It harbors some level of pre-existing tensile stress patterns, but ablation-derived stress patterns appear to generally dominate over the pre-existing stress as previously reported in [29]. Cells harbor relatively simple shapes, very rarely divide, and grow rather slowly. A single microtubule reporter is sufficient to extract the CMT arrays signal of interest as well as the cell contour signal. Finally, the generation time of the sample (In total 6 days after sowing the seed), is very practical in the context of relatively high-throughput screening.

With this workflow, we also introduce a new approach to objectively quantify the circumferential reorientation of CMT arrays around the ablation. We generate what we call a “proxy for tensile stress pattern,” simply using the geometry of the ablation as a starting point. Here it is important to reiterate that this is not an actual computational simulation of the mechanical stress patterns in the tissue, but simply a geometrical reference that we use as a proxy for the expected circumferential stress generated by the ablation. Indeed, while ablations are expected to generate a circumferential stress pattern, the tissue in which the ablation is performed is already under the influence of a pre-existing stress pattern. For instance, previous work has revealed that light-grown hypocotyl epidermis experiences longitudinal tensile stress associated with differential growth during elongation [29, 30]. Nevertheless, as mentioned above, the ablation changes the stress pattern around it and generally appears to override the pre-existing stress locally. A more advanced solution to predict stress patterns would be to generate finite element-based mechanical simulations of stress and strain for each specific case. However, this may require additional assumptions, since for instance a cylindrical tissue like the hypocotyl would be predicted to experience a twice higher circumferential than longitudinal stress, if considering only internal pressure-derived stress. A more accurate simulation would thus require adding longitudinal tension to re-create the effect of the differential growth and anisotropy of inner tissues. Such case-by-case simulation with specific assumptions of pre-existing stress patterns is not yet applicable for such a high-throughput image analysis framework. Our geometrical proxy, on the other hand, is very simple to implement, fast to run, and based on a simple assumption. We assume that the ablation-induced stress pattern largely overrides the pre-existing stress pattern as was previously reported [16, 29], while this may also vary based on specific cases, we believe that it is a good proxy for our purpose.

It is also important to consider the actual meaning of the values extracted by FibrilTool in our workflow. In particular, the anisotropy value quantified can be sensitive to the image quality, intensity, and background generated by cytoplasmic fluorescence signal [21]. While FibrilTool is the tool implemented in this workflow to quantify CMT array organization, other ImageJ plugins such as OrientationJ could be implemented instead in order to overcome limitations emerging in specific cases. The question of imaging resolution is also important when it comes to generate the most accurate quantification especially when imaging structures with diameters lower than the resolution limit of the imaging system, as is the case here. However, when doing timelapse imaging it is also important to find the good balance between acquisition time, and resolution to avoid problems with fluorophore bleaching or having acquisition time for a single stack being longer than the targeted time interval between images. In our case, we used a regular point-scanning confocal system which is the most widely available type of confocal system and decided to use a 40 × objective with a numerical aperture of 1, which provides us a good balance between field of view and resolution for this application. The theoretical resolution limit of the objective with the wavelength used in our case (Abbe diffraction limit) is approximately 0.29 µm for the lateral resolution (xy) and 1 µm for the axial resolution (z). Applying the Nyquist criterion for sampling rate would thus suggest using an acquisition resolution of about 0.1 (xy) and 0.4 µm (z) for optimal settings. With our microscope, our targeted field of view, and the average number of slices we acquire per stack, the acquisition time for a single time point would be in the range of 30–45 min, which is about twice as long as our target time interval, not to mention the amount of sample bleaching occurring during such acquisition time. We thus compromised by decreasing the resolution to 0.3 (xy) and 0.5 µm (z) which generally yielded a stack acquisition time of roughly 5 min. In our experience, this was sufficient to record and accurately quantify CMT arrays reorganization over time. However, depending on the specific case (size of the sample, cells, ablation, etc.) other objectives with other specifications may be more useful, or faster confocal technologies could be useful to reach the ideal resolution with very fast imaging and very little bleaching (e.g., resonant scanner, spinning disk or light sheet). The post-acquisition accuracy of the quantification with different resolutions is also a concern more specifically related to the image processing and algorithm of FibrilTool rather that this workflow in which other methods of CMT array organization quantification could be used. Yet, as discussed in the original FibrilTool publication, “Although this study validates the plug-in, the numbers […] should not be generalized, as they depend on the type of images analyzed. Therefore, an expert evaluation of the plug-in will always be required on a few images that are typical of the set to be analyzed” [21]. In the context of our workflow, it is now well established that CMT arrays in wild-type plants should undergo a significant reorganization over time (as measured with FibrilTool), such that being able to reproduce this observation could already serve as a good indication that the imaging parameters are adequate.

In the first step of this workflow, 3D stacks are directly projected in 2D thanks to the macro SurfCut2. Such 2D projection of 3D signal could in principle lead to the distortion of the signal that may exist in plans which are oblique to the 2D projection (e.g., cells on the side of the hypocotyl). However, epidermal plant cells are often largely convex at their surface such that they generally still have a large part of their cortical signal that exists in a plan parallel to the 2D projection and would thus not be majorly distorted (see illustration in Additional file 3: Supplemental Fig. S2). Furthermore, our workflow generates ROIs for the CMT analysis that exclude the edges of the cells and thus tends to exclude signal that is very oblique to the 2D projection (Additional file 3: Supplemental Fig. S2G). It is nevertheless advisable to analyze ablation performed on surfaces parallel to the 2D projection, and potentially exclude from the analysis cells which are obviously very much on edges and would have their signal largely distorted by a 2D projection. Note that due to the anisotropic nature of the confocal signal (point spread function; xy resolution higher than z), performing such analysis in 3D or on curved surfaces would also generate a strong bias in the quantification (Additional file 3: Supplemental Fig. S2A and B).

A potential source of variation in the response is the variability of size and shape of the manual ablations. In principle, larger ablations will generate more intense stress since stress intensity should be proportional to the radius of the ablation [16]. In turn, if the ablation is not perfectly circular, we could also expect variations in stress intensity along the perimeter of the ablation. A more accurate ablation method consists in using a pulsed laser to disrupt the cell wall and thus ablate specifically one or several cells [6]. If available such an approach should be used to generate ablation which is more precise and similar in shape and size. This would also allow better control over the timing between the ablation and the first time point acquired and as well as the possibility to easily record a time point before the ablation to compare the state of the CMT arrays before and just after the ablation. However, this requires specific equipment that is not broadly available. Conversely, manual ablations are very easy to perform even for beginners and require very little equipment, but the tradeoff is the variability of shape and size of the ablation and the delay between the ablation and the first acquired timepoint.

Finally, the ablation assay is only one of the methods that can be used to assess the mechanical stress response of the CMT arrays. Physical damages resulting from the ablation also induce chemical and hormonal responses that could influence the CMT response [31]. Thus, while our workflow now opens the possibility for high-throughput quantification of CMT response to tensile stress, which can be particularly useful in the frame of a forward or reverse genetic screen, new observations, and mutant characterization should then be further confirmed with alternative methods such as tissue compression or stretching [16, 30].

## Conclusions

Altogether, our new, open, and user-friendly workflow allows a high throughput and objective characterization of the CMT arrays response to mechanical stress. As such, it will be particularly useful to dissect the molecular pathways that govern the dynamic behaviors of CMT arrays such as those involved in the response to tensile stress patterns.

## Methods

### Plant material and growth conditions

The CMT reporter lines used in this study are *p35S::GFP-MBD* in WS-4 [25], *pPDF1::mCit-MBD* [26] and *p35S::GFP-TUA6* [27] in Col-0 background, and a katanin mutant line *bot1-7* (WS-4) in *p35S::GFP-MBD* [8]. These lines are referred to as GFP-MBD, mCit-MBD, GFP-TUA6, and *bot1-7* GFP-MBD respectively in the text. Seeds were sown on plates containing 1MS; 1% sucrose; 0.8% plant agar; 0.5 g/l MES; pH fixed at 5.7 with KOH. Once on the plates, seeds were cold and dark treated for 48 h to synchronize their germination. Seedlings were then grown in vitro in a growth chamber under long-day 16-h/8-h (light/dark) period at 22 °C.

### Ablation procedure

Seedlings were arranged with minimal perturbation and immobilized with 2% low melting agarose (TopVision, Thermoscientific) on the agar plate on which the plants were grown. The ablation was performed as in [29], under a binocular with a fine Minutiens needle (0.15 mm diameter) set on a pin holder to apply a small pressure at the surface of the hypocotyl to physically rupture a few cells (Supplemental Fig. S5). For an experiment, in general, 15 to 20 seedlings were placed on the plate and ablation was attempted for each sample. Due to the variability in precision of manual ablation, some samples did not appear to have ablation while others had ablations that were too large. Only those that appeared to have resulted in the ablation of approximately 4 cells were kept for imaging and further analysis. In the case of the mock experiments, the samples were prepared as described above without performing the ablation.

### Confocal image acquisition

Once the ablation was performed on the hypocotyls, the plate was set under an upright confocal microscope to be imaged using a long-distance water-dipping objective (Additional file 3: Supplemental Fig. S3B). Images were acquired 5 to 10 min after performing the ablation, with a Zeiss LSM 800 upright confocal microscope using the Zen blue software for the acquisition. A 40 × water dipping objective (W Plan-Apochromat 40 × /1.0 NA) was used. Z-stacks were acquired without averaging and with a 0.5 µm Z step in time series every 20 min for 4 h. The images have a size of 1024 × 512px for a pixel size of 0.31 µm. For all tested lines, samples were excited with the 488 nm wavelength, and the emission was collected in the range of 400–530 nm for GFP signal and 400–550 nm for mCit signal. The laser reflection was filtered by a beam splitter built in the LSM800.

We acquired timelapses for nine samples (biological replicates) for each genotype and each condition (with and without ablation) for a total of 72 timelapses, each containing 13 timepoints 3D stacks. The exact number of cells analyzed in each case is reported in the legend of Fig. 2.

### Analysis of CMT arrays

The workflow is based on the Fiji distribution of ImageJ. The macros described here can be downloaded from GitHub (https://github.com/VergerLab/MT_Angle2Ablation_Workflow) [17] and Additional file 1. The overall concept and procedure are described in the main text (Fig. 1) and a detailed step-by-step description of the procedure is available in the user guide available in the GitHub repository and as Additional file 2.

In the case of time series on intact hypocotyls, the image analysis procedure was the same as described above. An area consisting of approximately four cells was defined as a mock ablation and the CMT arrays were quantified in the cells around the mock ablation (see Additional file 3: Supplemental Fig. S6, S9, S12, S15, B).

All intermediate processing data generated by the workflow for the analysis reported in this paper (SurfCut projections, cell contour preprocessing, ROIs, geometry-based proxy, FibrilTool output, and angle to ablation quantification) have also been deposited at https://zenodo.org/record/7436075#.Y5rmd-zMJF8 [32].

The newly generated macros are mainly made of built-in ImageJ macro functions using the ImageJ macro language. In addition, in the macro “TmlpsCellContour_Preprocessing” (tool named “Cell Pre proc”), we use the “Linear Stack Alignment with SIFT” plugin (pre-installed in Fiji) for the affine registration. In “SimuAblation_Cell_RoiMaker_timelapse” (tool named “Roi Maker”) we use the “Linear Stack Alignment with SIFT MultiChannel” (“PTBIOP” update site) for multichannel rigid registration. In both “TmlpsCellContour_Preprocessing” and “SimuAblation_Cell_RoiMaker_timelapse,” we use the “Mophological Segmentation” and/or the “Interactive Marker-controlled Watershed” segmentation as well as the “Region Adjacency Graph” process from the MorphoLibJ plugin (“IJPB-Plugins” update site; [20]. The exact parameters used in these functions can be found and modified if needed in the macro codes [17] (https://github.com/VergerLab/MT_Angle2Ablation_Workflow).

For the generation of the geometry-based proxy for the circumferential tensile stress orientation, our workflow simply makes use of the ablation shape segmented during the cell segmentation process. More specifically, the cell contour image is segmented with MorphoLibJ which generates individual labels for each segmented area (cells, ablation, or background; Fig. 1F). From this segmentation, the user is prompted to select the label corresponding to the ablation site. This label is then extracted in a separate image and converted into a mask which is then eroded 3 times with a radius of 1 pixel. This mask is then converted into an ROI (“Analyze particles…” function of ImageJ) and saved as a “.roi” file. A black 8-bit image of the same xy dimensions as the acquired data is then generated and the ablation ROI is loaded on this image. The ROI is then enlarged (“Enlarge…” ImageJ function) with a given value (“spacing value” input in the macro prompt). The new enlarged ROI is then converted into a band (“Make band…” ImageJ function) of 1-pixel width, which is then used to draw a white line (“Fill” ImageJ function) on the black background of the 8-bit image. This process is repeated several times (“iteration number” input in the macro prompt). Finally, the 8-bit image with white lines is saved and will be later-on analyzed with FibrilTool as the proxy for tensile stress orientation. Note that the ablation shape can also be first converted into its convex hull (“Convex Hull” ImageJ function) before running the enlargement and line drawing process. This can be done by changing the value of the variable “AblConvexHull” from “false” to “true” in the macro code (see user guide).

The "FibrilTool_Batch_Workflow" macro (tool named “FibrilTool”), is a modified version of the macro “FibrilTool_Batch.ijm” [22], which was a modification of the original “FibrilTool” Macro [21], allowing as input a Zip file containing ROIs for batch processing of several ROIs in a single image. Our modification further allows the integration into our workflow with a batch mode over several images in a folder and allows users to choose between processing images from actual CMT arrays or corresponding images from our geometry-based proxy.

### Statistical analysis

A python-based data processing script was developed and is accessible within a Jupyter notebook (https://github.com/VergerLab/MT_Angle2Ablation_Workflow). Plots and tables are generated using the python library Pandas and Seaborn and represent the mean and bootstrapped (*n* = 1000) 95% confidence interval (as calculated by the seaborn library). Note that the bootstrapping process uses random resampling of the population such that each new calculation of the bootstrapped 95%CI generates a very slightly different output. Data reported in the text are rounded to whole numbers for angle values and at two decimals for anisotropies.

## Supplementary Information

**Additional file 1.** ImageJ macro file containing the workflow code: Angle2ablation_Workflow_ToolSet.ijm.

**Additional file 2.** Workflow’s step-by-step user guide.

**Additional file 3: Figure S1.** High-throughput image analysis workflow to quantify CMT arrays in pavement cells. **Figure S2.** 3D vs. 2D context and rational for the analysis of projected signal from curved samples. **Figure S3.** Sample preparation and experimental design. **Figure S4.** Comparison of workflow output using manual estimation vs. geometry-based prediction of tensile stress pattern around the ablation. **Figure S5.** Plots of time series with different time resolutions. **Figure S6.** Time-lapse of the GFP-MBD reporter line after ablation or mock experiment. **Figure S7.** Individual sample plots of CMT arrays for the GFP-MBD reporter lines. **Figure S8.** Individual cell plots of CMT arrays for the GFP-MBD reporter lines. **Figure S9.** Time-lapse of the katanin mutant *bot1-7* GFP-MBD reporter line after ablation or mock experiment. **Figure S10.** Individual sample plots of CMT arrays for the katanin mutant *bot1-7* GFP-MBD reporter lines. **Figure S11.** Individual cell plots of CMT arrays for the katanin mutant *bot1-7* GFP-MBD reporter lines. **Figure S12.** Time-lapse of the mCit-MBD reporter line after ablation or mock experiment. **Figure S13.** Individual sample plots of CMT arrays for the mCit-MBD reporter lines. **Figure S14.** Individual cell plots of CMT arrays for the mCit-MBD reporter lines. **Figure S15.** Time-lapse of the GFP-TUA6 reporter line after ablation or mock experiment. **Figure S16.** Individual sample plots of CMT arrays for the GFP-TUA6 reporter lines. **Figure S17.** Individual cell plots of CMT arrays for the GFP-TUA6 reporter lines.

**Additional file 4: Table S1.** Mean Angles. **Table S2.** Mean Anisotropies. **Table S3.** All_Data.

## Data Availability

All the microscopy data generated and analyzed for this study have been deposited at the Swedish National Data service (https://doi.org/10.5878/17te-jg54) [33]. All intermediate data generated from processing steps (processed images, regions of interest, quantifications, logs, etc.) are available at (https://zenodo.org/record/7436075#.Y5rmd-zMJF8) [32]. The final raw data file output from the quantification used to generate the plots is also available in Additional file 4 under Supplemental Table S3. The code newly generated for this study is available at https://github.com/VergerLab/MT_Angle2Ablation_Workflow [17].
