# Supplementary material for: High-throughput characterization of cortical microtubule arrays response to anisotropic tensile stress
Source: BMC Biol. 2023 Jul 10;21:154. doi: 10.1186/s12915-023-01654-7 (PMC10334548; doi:10.1186/s12915-023-01654-7)
Supplement: Supplementary file 2 — Additional file 2. Workflow’s step-by-step user guide. [file 12915_2023_1654_MOESM2_ESM.pdf]

Step-by-step user guide for the analysis of cortical microtubule response after ablation

MT_Angle2ablation_Workflow

Procedure overview and quick guide

Here we describe step-by-step how to operate the image analysis workflow we developed to quantify cortical microtubule arrays reorganization in plant cells following a mechanical stress induced by an ablation. The workflow requires several components under the form of imageJ macros and a python Jupyter notebook for the data analysis:

Load (**>>**) "Angle2ablation_Workflow_ToolSet" in Fiji and run each macro as needed:

1) Run "SurfCut2" to a) extract cell contours and b) the outer epidermal cortical microtubules (CMTs) signal.
2) Run "Folder Maker" to automatically generate a folder architecture to organize your data.
3) Run "Cell Pre proc" to enhance the cell contour contrast around the ablation and improve automated segmentation.
4) Run "ROI Maker" to define the ROIs used for quantification and generate a geometry-based prediction of the tensile stress pattern around the ablation.
5) Run "FibrilTool" to a) quantify the microtubules organization in each cell ROI previously defined as well as b) to quantify the average orientation of the tension according to the geometry-based proxy.
6) Run "A2A" to calculate the angles of CMT arrays relative to the predicted tension.
7) Run "Stats" which will guide you to the online version of the "A2A_Tmlps_Stats" python notebook to process the data and generate graphs.

See below for the details step-by-step guide.

Prerequisites

Software

- Fiji (https://fiji.sc/)

Additional plugins required:

- MorphoLibJ ("IJPB-Plugins" update site)

- Linear Stack Alignment with SIFT MultiChannel ("PTBIOP" update site)

Workflow scripts:

- Angle2ablation_Workflow_ToolSet.ijm (https://github.com/VergerLab/MT_Angle2Ablation_Workflow)

- A2A_Tmlps_Stats.ipynb (https://github.com/VergerLab/MT_Angle2Ablation_Workflow)

Data:

Note regarding image acquisition: any fluorescent microtubule reporter line can be used to acquire Z-stacks with a confocal microscope at different time points after applying a mechanical stress. In many cases, the CMT signal is sufficient to also be used as a cell contour signal, but in certain cases it could be necessary to also acquire a second channel specifically for cell contour signal. For a better-quality analysis, it is recommended to use z-intervals of at least 1µm.

Installation

For more details see the installation page on the github repository.

- Fiji: see https://fiji.sc/

- Plugins: To install the required plugins, turn on the corresponding update sites. See https://imagej.net/update-sites/following for explanations if needed.

- Angle2ablation_Workflow_ToolSet.ijm: Download the “code” from the GitHub page (https://github.com/VergerLab/MT_Angle2Ablation_Workflow).

Click on 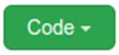

around the top right corner of the page, and then ”download zip”. Then, unzip the file in the folder of your choice. Finally, copy the Angle2ablation_Workflow_ToolSet.ijm file and past it in the macros/toolsets folder of your Fiji install folder (on a Mac, access this by right clicking on the Fiji app in a Finder window and selecting "Show Package Contents").

To check if the toolset was loaded properly, open Fiji, and click on 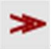 at the right end of the Fiji window. You should see Angle2ablation_Workflow_ToolSet in the drop-down menu. Select it and the toolset should appear in your Fiji toolbar.

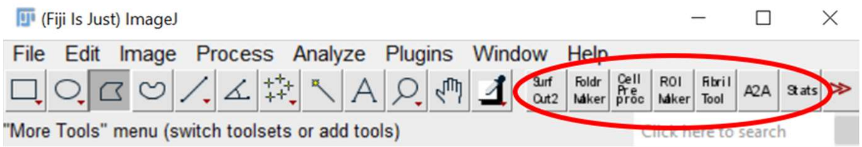

- A2A_Tmlps_Stats.ipynb: No installation is required as the notebook can be run online simply by clicking on the 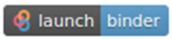

button on the github page. For local installation of the notebook see the more detailed installation procedure at https://github.com/VergerLab/MT_Angle2Ablation_Workflow.

Workflow:

First, after image acquisition, we advise keeping a separate folder with all the raw untouched data and create a separate folder to convert and save all individual acquired stacks in .tif format (look online, there are many macros that exist to do this task in batch for all your images, and different format of raw images). **Each 3D stack should be saved separately, not as a timelapse hyperstack.**

1) SurfCut: Cell contour and CMT signal extraction

a. Following the information provided in the SurfCut user guide (https://github.com/VergerLab/SurfCut2), extract the cell contours and the outer epidermal CMT signal (This case is specifically covered in the user guide).

b. Make sure to specify suffix for the cell contour images as "_cells" and "_MTs" for the CMTs images when saving them. These suffixes will be recognized by the macros used later on. Note that there is no need to save the Original Projection or the SurfCut Stack, but only the SurfCut Projection.

SampleName_1_cells.tif

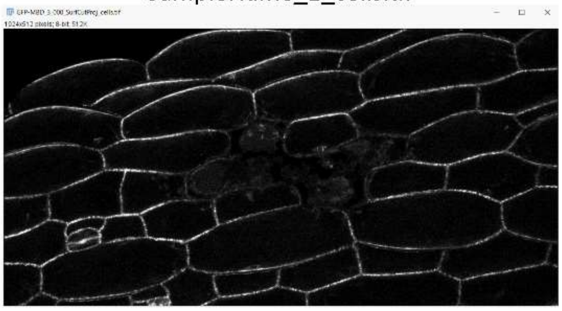

SampleName_1_MTs.tif

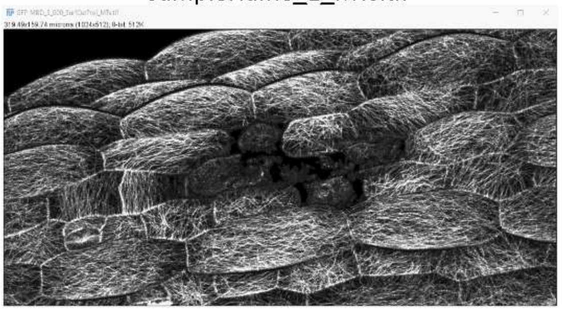

2) Folder maker: Generate the folder architecture according to your samples and conditions (optional)

For this workflow, following SurfCut processing, we advise using a specific folder architecture (Experiment/Genotypes/Samples), that then allows the last steps "A2A" to be run over the whole experiment to generate a single text file containing all the data for further statistical analysis.

a. Click on the "FoldrMaker" tool. In the pop-up window, specify the chosen experiment name, location where the folder should be created, name the different genotypes/conditions and the number of samples per genotype/conditions. For genotypes/conditions names, make sure to follow the nomenclature specified in the examples (Names separated by a coma and a space, and of course remove the parenthesis and "e.g."). Then click Ok.

b. After the folders are created, copy the images that have been extracted with SurfCut in step 1, in their corresponding folders.

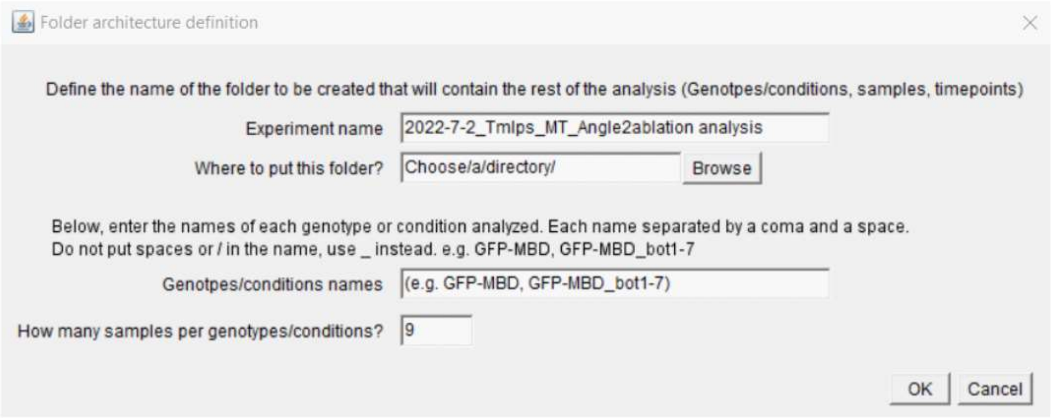

3) Cell Pre proc: Preprocessing for cell contours improvement (optional)

Before running this tool make sure that "Linear Stack Alignment with SIFT MultiChannel" and "MorpholibJ" plugins are installed.

"Cell Pre proc" is a tool to prepare the segmentation of the cells around the ablation. This tool aligns all images from one time lapse and runs a z-projection using the average intensity. This image will be used to optimize the cell segmentation of the analyzed sample.

a. Click on "Cell Pre proc" in the tool menu. First, we advise running the tool on one sample (time series), to test the parameters, before applying them to all.

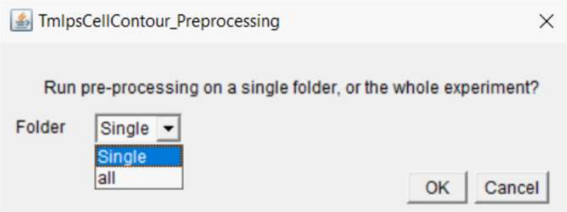

b. You will then be prompted to choose a directory. In this case, choose a "sample" folder: a folder containing all the images of a time series for a single sample).

c. In the next step, choose how many images to use for the alignment (starting from the first time point). Note that in some cases it is better to align only the first few images of the time lapse depending on the sample. Over time, the morphology of the sample can change bending/twist of the hypocotyl; this can create artefacts during registration and average projection and render lower quality cell contours.

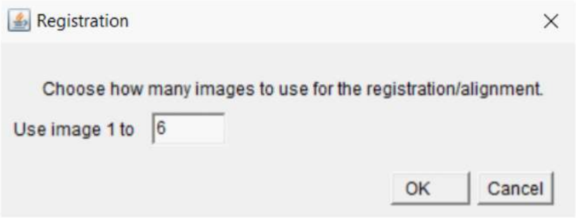

d. To test the quality of preprocessing for further image segmentation, you can then test the morphological segmentation from MorphoLibJ directly from within this macro. This segmentation is simply a test that will not be saved.

e. Finally, you can run the tool on all samples at once. You can then check the result for each time series and go back to processing/make corrections for some of them one by one with the “single” mode if necessary. This will simply overwrite the previous preprocessed image.

4) ROI Maker: Define regions of interest and geometry-based tensile stress pattern prediction.

This macro contains several steps, from cell segmentation, ablation definition, ROI creation, ROI refining, and geometry-based tensile stress prediction. These are put together because some of the same segmentation data are used both for the creation of refined ROIs and the tensile stress pattern prediction.

Note that the geometry based tensile stress proxy is generated form the segmented shape of the ablation which can sometimes be locally concave. In some cases, it can be useful to transform this shape with a convex hull. This is possible by simply changing one parameter in the macro code at line 1044. Open the “Angle2ablation_Workflow_ToolSet.ijm” file from the macros/toolsets folder of your Fiji install by dragging and dropping in Fiji. In the script editor, replace the value of “AblConvexHull” form “false” to “true”. Save the file and reload the ToolSet (>>).

```
1040 ///==========Input/output file names parameters==========///
1041 // Input paramaters: input files suffixes
1042 contours_suffix = "_cells.tif";
1043 features_suffix = "_MTs.tif";
1044 AlbConvexHull = false; //true
1045
1046 // Output paramaters: output file suffixes
1047 contour_Roiset_suffix = "_RoiSet_cells.zip";
1048 Ablation_Roi_suffix = "_Ablation.roi";
1049 Simu_MT_suffix = "_MTs_Simu.tif";
1050 original_contours_suffix = "_cells_original.tif";
1051 original_features_suffix = "_MTs_original.tif";
1052
1053 ///======================================================///
1054 ///======================================================///
1055 ///======================================================///
```

a. Click on the “ROI Maker” tool. In this case, each samples folder is processed one at a time. In the pop-up “choose a directory” window, choose a “sample” folder: a folder containing all the images of a time series for a single sample).

b. First the macro will open all the time points in the time series and align them (with rigid registration; no distortion of the image). Go through the stack to check if the cells of the sample in the time lapse stack and the time lapse alignment stack are visible throughout all the time points and if there is no problem with the images. Then validate.

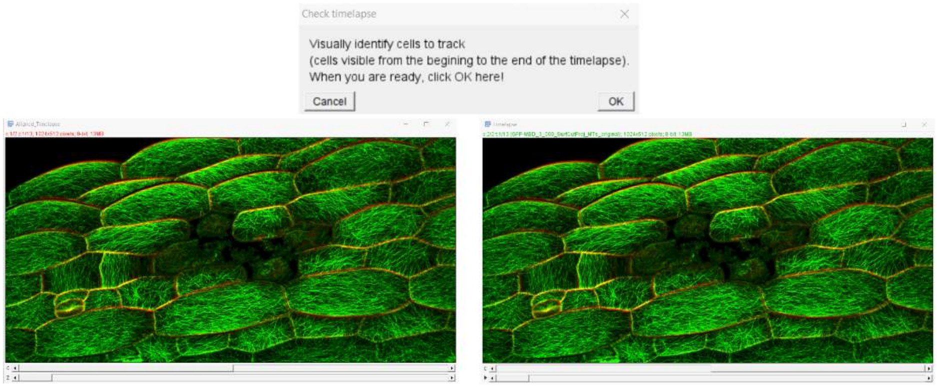

c. Next, the image obtained with the “Cell preprocessing” tool (or the first cell contour image of the time series) will pop-up with a dialog box to choose a segmentation method. There is a choice between “Morphological” (fully automated) and “Marker watershed” (markers need to be placed manually in each cell) segmentation.

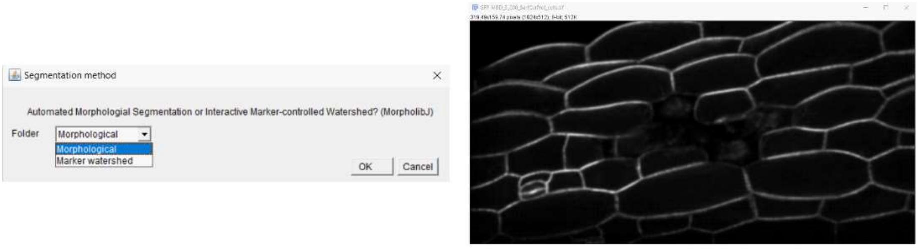

The most straight forward approach is to use the “morphological segmentation” if cell contours are clear (steps d-e. If the result is not satisfying, then switch to marker assisted segmentation (d’-e’).

d. The “Morphological” segmentation method opens the segmentation window. The segmentation runs automatically with default parameters. The Watershed segmentation has a tolerance of 10 but this can be modified until the segmentation fits. In the example below the result is not satisfactory. The options are thus either to change the tolerance parameters and re-run the segmentation until the segmentation looks ok, or switch to the marker-based segmentation (see next step).

Note that here on the image below there is clearly some over-segmentation. In some cases, this is acceptable, and can be corrected in a following step by merging some labels.

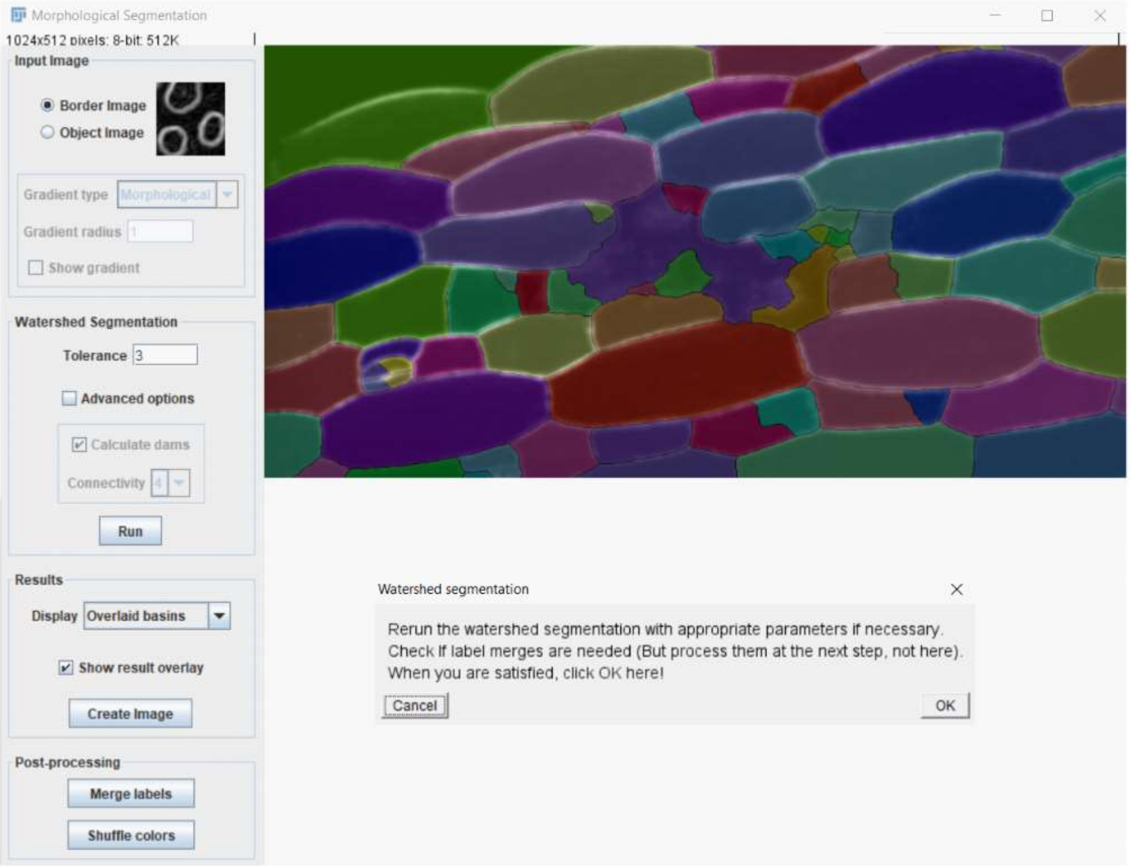

Below is an example sample for which the automated segmentation worked properly.

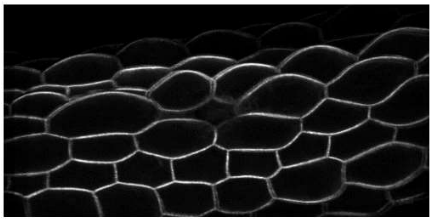

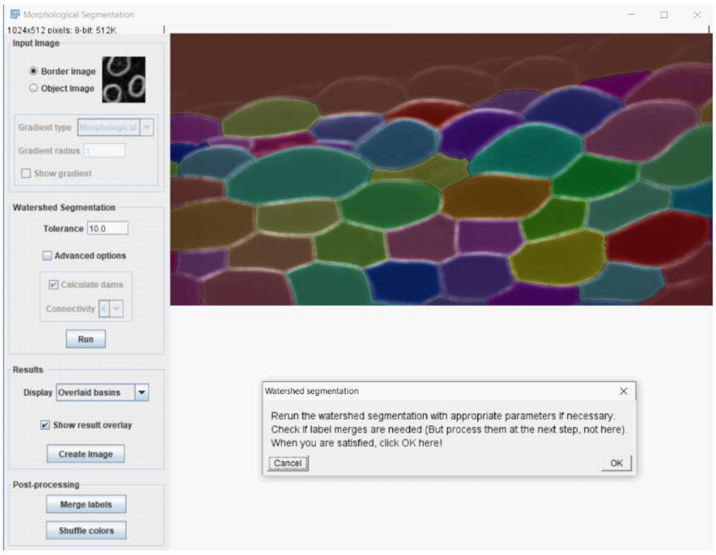

e. In the dialog box that pops up next, select “Yes” and “OK” to continue the analysis. If not, select “No” and “OK” to return to the segmentation method choice.

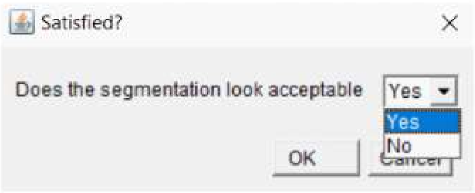

d'. If you chose “Marker watershed” segmentation, a segmentation window opens. Select manually by using the “Multi-point” tool, the ablation and the cells around it. Note that it is better to select at least two layers of cells around the ablation.

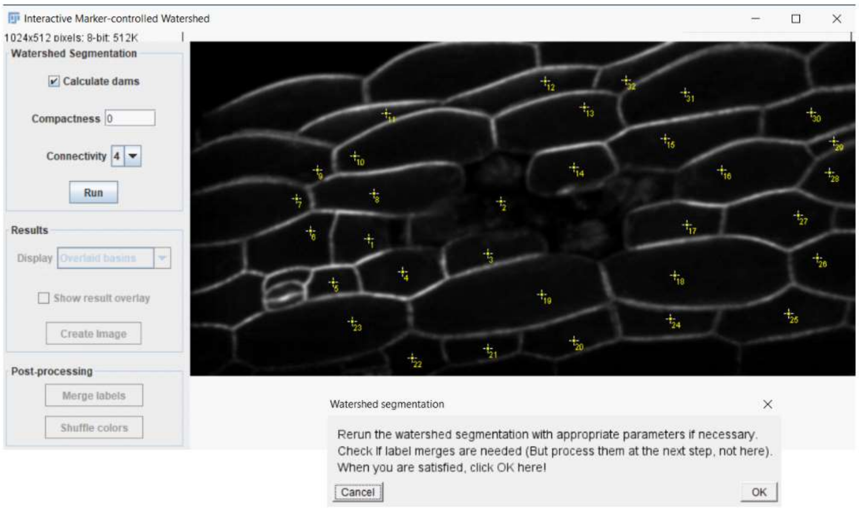

e'. Click on “Run”. In the result below, there is some under segmentation. One possibility is also to change the position of some markers to see if it improves segmentation or add additional markers and fuse some labels later on.

If none of this fully works for all the cells, there is a possibility in a later step to draw/correct manually some of the ROIs that will be created from these segmentations (see step h.).

If satisfied, click on “OK”. The same dialog box as before will appear. If the segmentation is good click on “Yes” to continue the analysis.

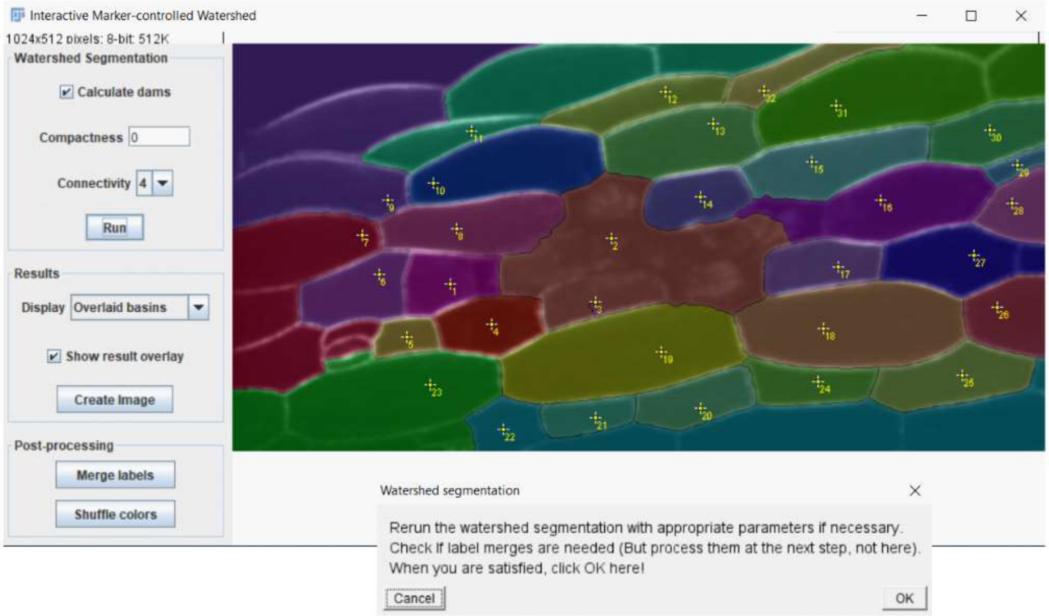

f. During the next step you can correct some of the segmentation errors by merging some labels. Follow the instructions in the pop-up window.

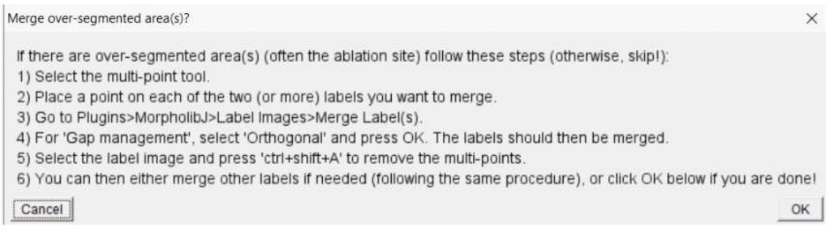

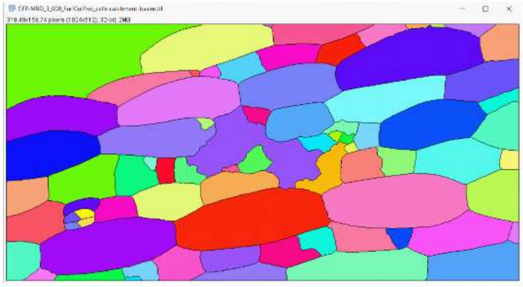

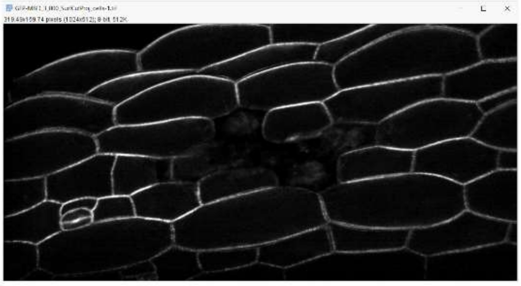

In this case, below is the expected result after correction. Not all segmentation errors have been corrected, only those for the ablation and the cell layer around the ablation.

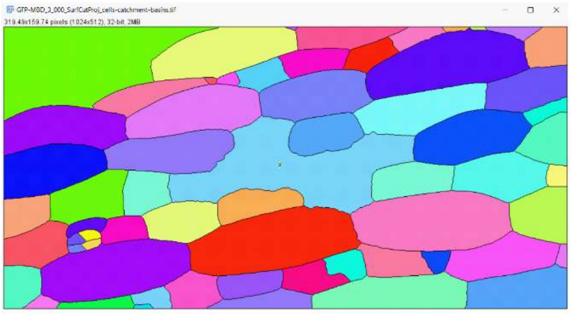

g. In the next step, you are prompted to select the ablation site. Follow the instructions in the pop-up window.

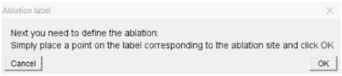

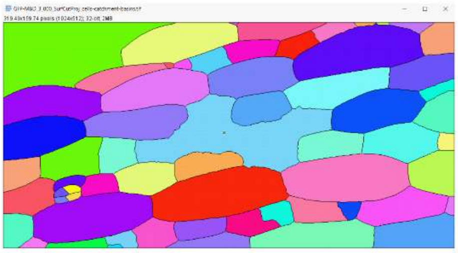

h. Next, the _MTs image appears with the ROIs, including the ablation and a single layer of cells around the ablation. In this step you can further correct the ROIs by deleting and re-drawing some manually if needed. To delete a ROI, select it in the ROI manager and press “Delete”. Then select the polygon tool, draw the new corrected ROI, and add it to the ROI manager (Click “add” or hit ctrl+T).

Note that the ROI of the ablation should be the first one on the list of ROI for the next steps of the processing. If you don’t modify it will stay as is, but if you delete it and re-draw it, the new ablation ROI will be the last one on the list. Then you need to manually rename the ROI with “0000000001” to make sure that the ablation ROI is first on the list. After renaming the ROI, click on “More” and select “Sort” to refresh the ROI list.

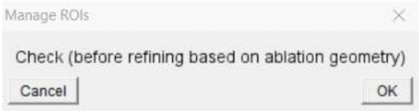

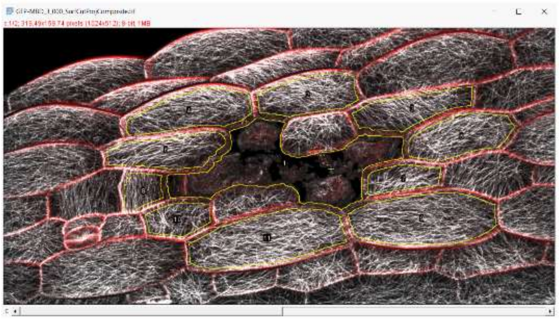

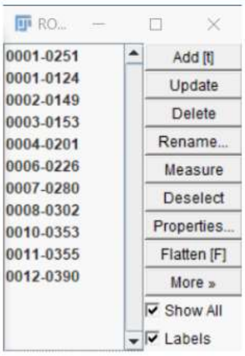

i. Next, the macro will create a simulation of tensile stress pattern around the ablation across the neighboring cells. Here you set a value for a “diameter”, which can vary depending on what you expect to measure. In our analysis we selected a value which is roughly the width of the cells in this tissue.

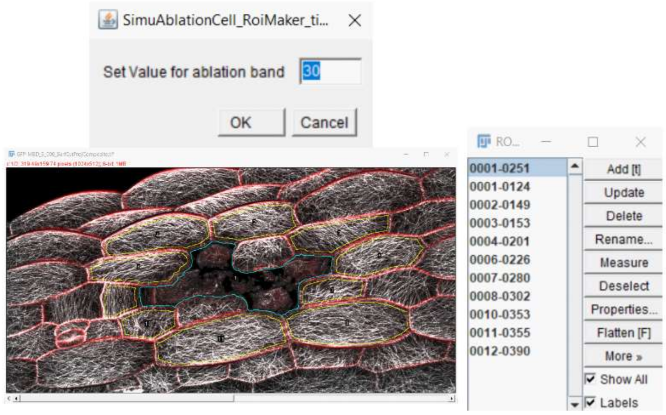

j. Below you see the outer edge of the “simulation” (larger ROI). This perimeter will be used to crop the ROIs of the cells surrounding the ablation. If satisfied click on “Satisfied” and “OK” or if you are not satisfied and then only click on “OK” and you will return to the previous stage where you can change the value until you are satisfied. Make sure to keep a constant value across your experiment and the samples that you want to compare afterwards!

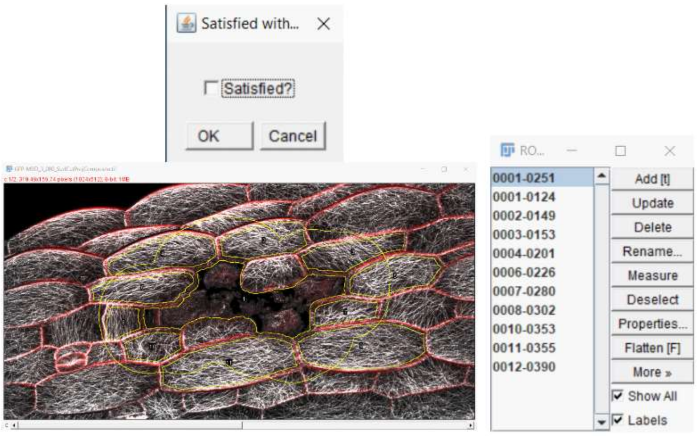

k. Next, the ROIs will be cropped according to the defined perimeter. Click on “OK” to continue the analysis.

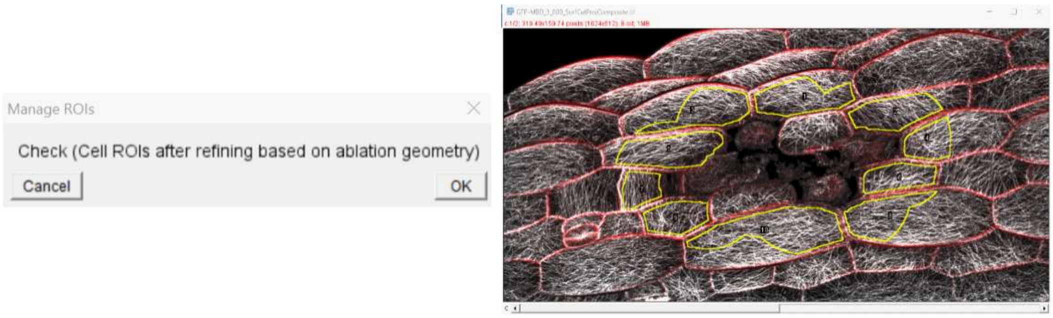

l. Next, you will define the parameters for the tensile stress “simulation”. Define the number of iteration (number of lines drawn) and the spacing between the lines.

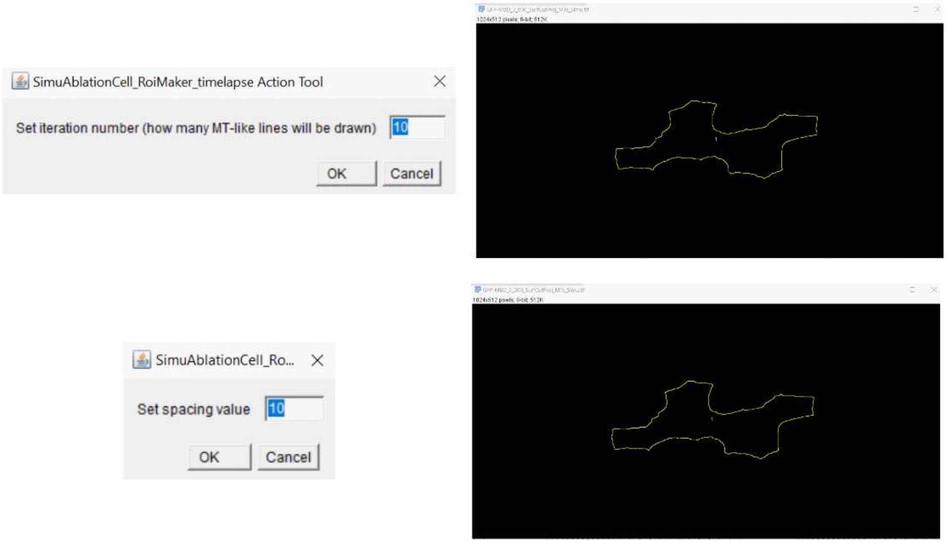

m. You can then check the simulation image to see if it is equally spread in each ROI. As always, you have the possibility to go back to the previous step if not satisfied.

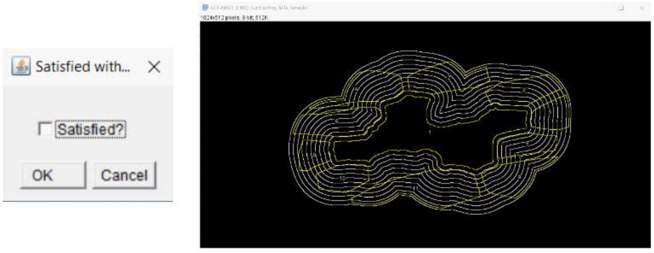

n. In this step, check the cell ROI positions for each time point of the time lapse. Sometimes there is a slight shift despite the alignment of the images in the time lapse. So, make sure the ROI is perfectly positioned in between the cell contours. Each time you click “OK”, the next image in the time series will open. Each time this will save the corresponding ROI files with corrected positions.

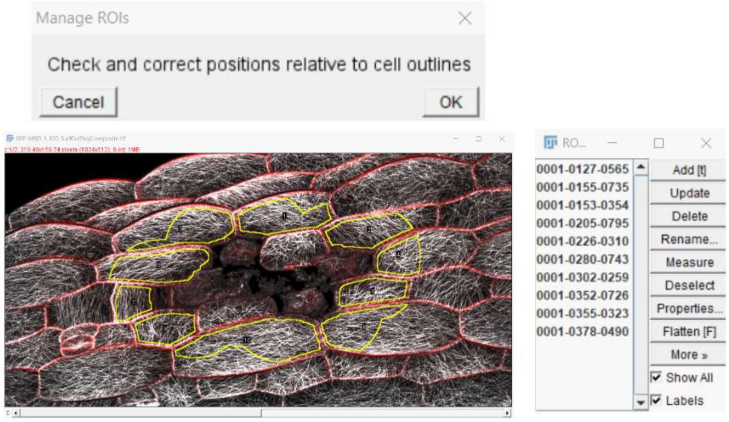

o. Finally, the last pop-up window leaves you the choice to stop processing samples for now or to continue directly with the same processing step for another sample.

5) FibrilTool: Microtubule arrays and predicted tensile stress patterns quantification.

This tool quantifies CMT arrays organization in each cell ROI previously defined, and outputs both angle orientation value (average orientation of CMTs (or tensile stress) and an anisotropy value. This version of the tool is adapted from FibrilTool_Batch (https://zenodo.org/record/2528872).

a. Click on the "FibrilTool" tool. In this case, each sample folder is processed one at a time. In the pop-up "choose a directory" window, choose a "sample" folder: a folder containing all the images of the time series for a single sample).

b. Next, a FibrilTool window opens to allow you to choose some parameters. These are mostly to change the result display on the output image and do not affect the analysis (lines color, numbering...). Here when you start FibrilTool, run the analysis first by selecting "Real" in the "Target for fibrils". This will perform the quantification on the actual CMT images.

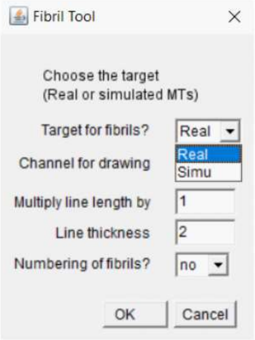

c. Once this is done repeat the same steps, but this time select "Simu" in the "Target for fibrils".

Below are example results images for both analysis with the visual FibrilTool output displayed as red lines.

"Real"

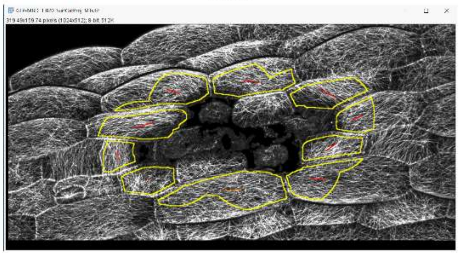

"Simu"

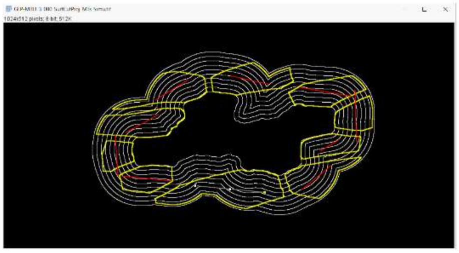

d. All data is automatically saved as txt files. Then repeat these steps for each sample.

6) A2A: Calculation of the "angle to ablation".

This macro calculates the "acute absolute angle" (between 0 and 90 degrees) between the actual microtubule array main orientation and the orientation of the predicted stress as measured with FibrilTool.

a. Run the “A2A” tool. In the choice for target, select “Simu”.

Note that here we leave the possibility to use manually drawn lines for reference of the tensile stress pattern, instead of measurements made on the geometry-based simulation, as was done in some previous studies. See towards the end for some details on how to use the “Line_ROIMaker macro”.

b. In the choice for folder, you can either choose “single” and this will run the calculation for a single sample (folder selected in the next step), or “all”. To run on “all” folders in the experiment (see file architecture generated at the beginning), all the samples must have already been processed in the previous steps. In this case select the Experiment folder containing all the genotypes/samples/… for this processing step.

Note that the “all” option will also generate a single text file containing all the quantification over the experiment, to be used for data analysis with the python notebook in step 7.

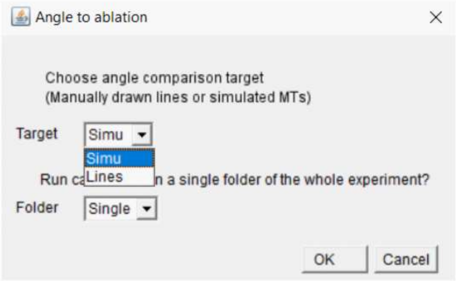

c. Next, it generates an image output and two files for each image from the subfolder containing the measurements (FibrilTool Angle; Drawn Line Angle; Raw angle; Absolute angle; Acute absolute angle to ablation; FibrilTool Anisotropy) and a file containing all the measurements of all the images in the subfolder. The image output (see below) with the angles written (yellow) on the image combined with FibrilTool output for the CMTs (Magenta) and the simulated stress pattern (green). This image is useful to confirm visually that there is no apparent mistake in the angle quantification.

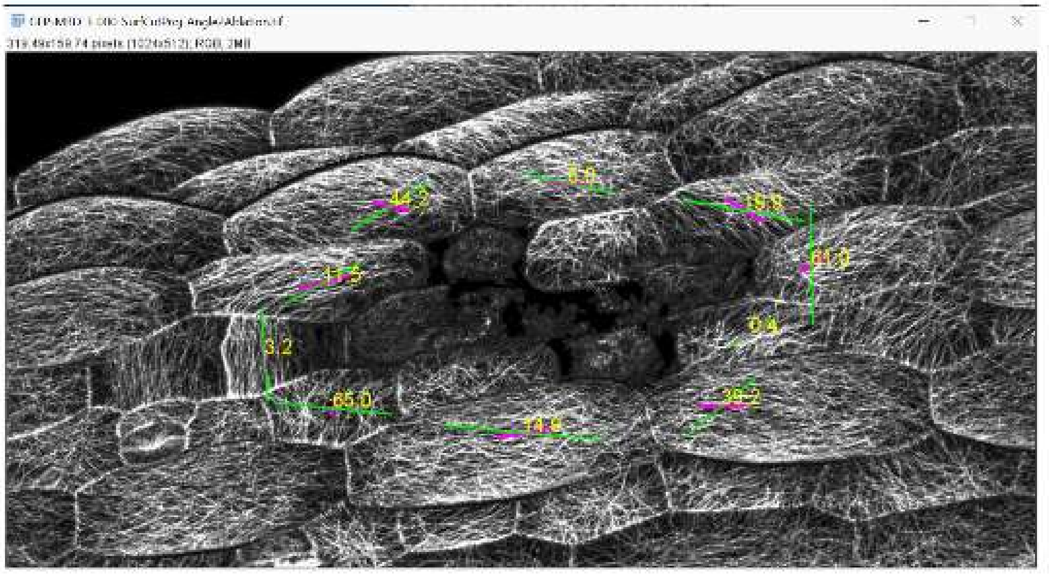

7) A2A_Tmlps_Stats.ipynb: Data analysis and plots.

Note that for this step, you need to have run the previous "A2A" analysis on "all" the folders at once (see step 6b) in order to generate the single txt file containing all the data of the experiment that will be used for this analysis.

The rest of the data analysis is done using a python script. All the information on the installation and use of python, jupyter notebook can be found in the Installation procedure.

Briefly, you can either run the python script with no install, directly on a web browser, or with a local install (see installation procedure on the github page).

a. To run it online with no install, simply go to the github repository page of this workflow (https://github.com/VergerLab/MT_Angle2Ablation_Workflow), and click on the "launch binder" button.

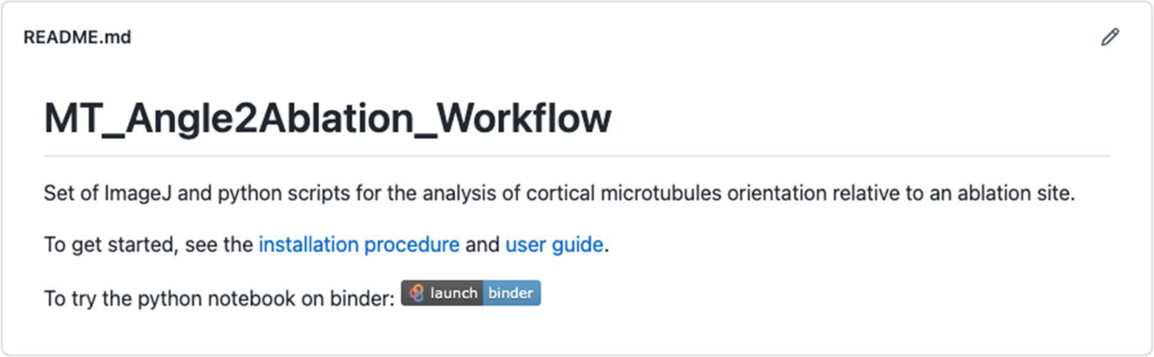

b. It will take a few seconds to process before the following page appears.

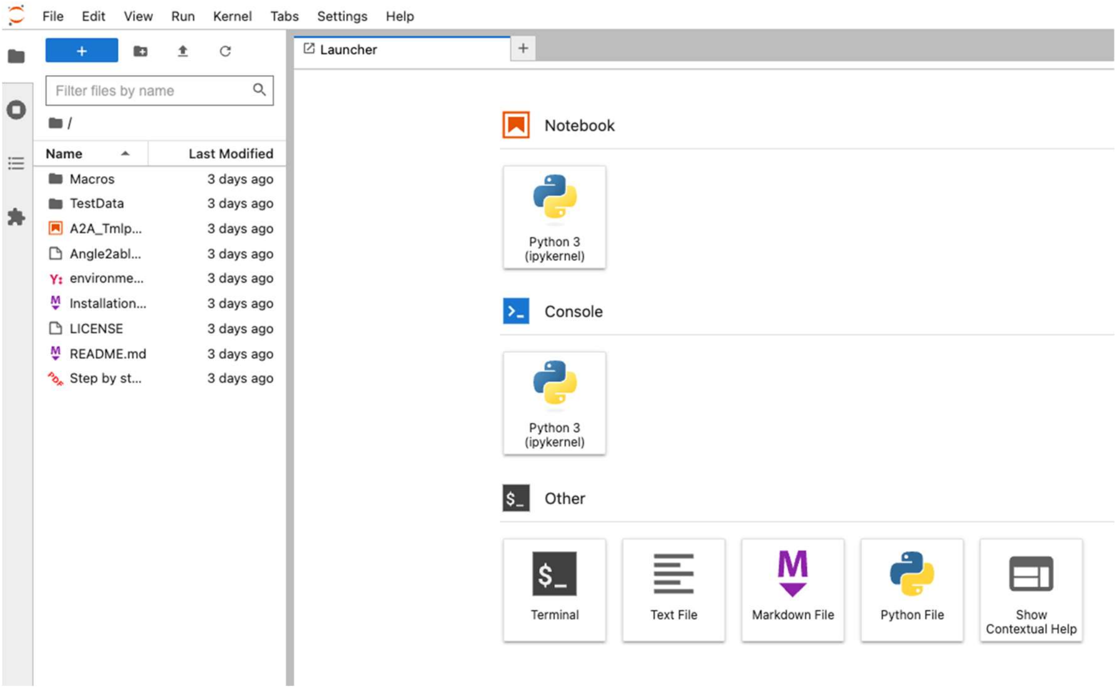

C. On the left, double click on “A2A_Tmlp...”. This will open the notebook. You can then follow the instructions in the notebook.

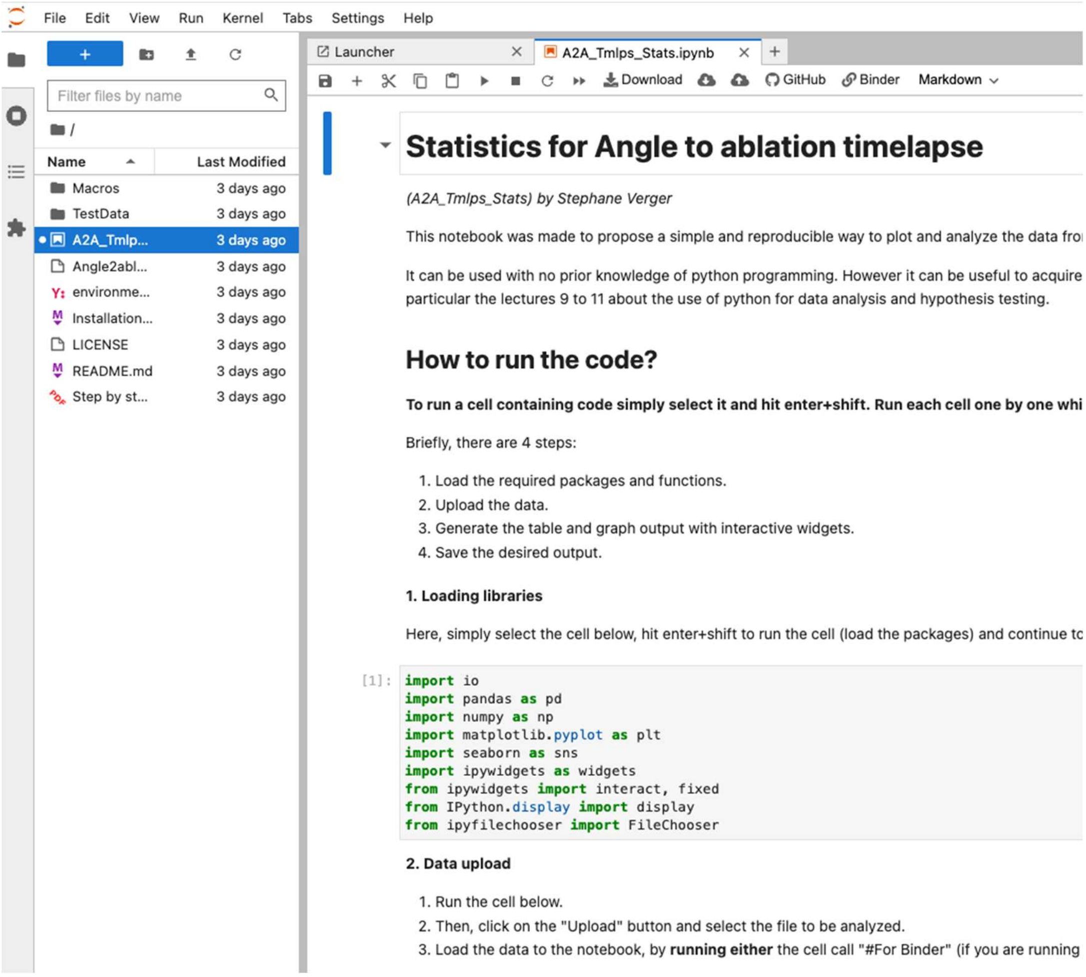

As explained in the notebook itself, you will first have to load the single data file generated at the end of the A2A macro when run on all samples. Then thanks to interactive widgets you will be able to display the dataset and make graphs. Finally, you will be able to save the data table of the values plotted as a .csv file, and the plots as .svg files.

Note that when running the notebook online, the data can only be saved on the temporary binder folder that is created to run the notebook online. In the cell where you choose the path for file saving, navigate to /home/Jovyan/. The data saved in the folder will appear in the file browser on the left of the notebook on this page. You can double click on it to open it in a new window there, or right click and select download to download it on your computer.

The .csv file can be opened in any text and spreadsheet editor. For Microsoft excel, first open a blank Excel workbook, then select the Data tab, click Get Data > From File > From Text/CSV (or search online for the specific system/version you use).

The .svg file can be opened and further modified (e.g. to change the time point naming) with any vector graphic editor (e.g. Inkscape or Illustrator).

(Optional/alternative step 4) Line_ROIMaker: Line representing the ablation stress for each cell with "Line_ROIMaker.ijm" (optional step)

Alternatively, to the geometry-based stress pattern prediction, you can use manually drawn lines to indicate the expected stress pattern around the ablation. This is the approach that has been used in previous studies. An ablation in a (ideally isotropically) tensed tissue is expected to generate a circumferential tension around the ablation site. In this case, to approximate this expected stress pattern, our strategy is to trace a line along the side of the cell facing the ablation. To limit user induced bias this line should be drawn between the two edges of the cell facing the ablation site (see figure below in c.).

You will first need to load the separate "Line_ROIMaker_Timelapse.ijm" macro. You can find it in the MT_Angle2Ablation_worflow folder under Macros/Deprecated.

a. Drag and drop the macro file in Fiji and run the macro.

b. Select the Straight-line tool in imageJ.

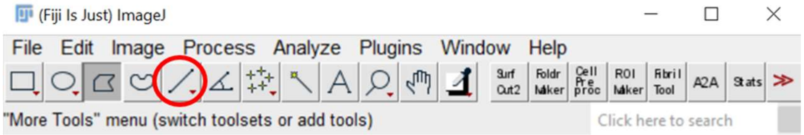

c. Draw manually each line corresponding to each cell in the image. Here it is important to note that each line ROI number must correspond to the cell ROI number. In the image, the cell ROI numbers are displayed. Start by drawing the line that corresponds to the cell (ROI) number 1 and add it to the ROI manager (Click "add" or hit ctrl+T). Then draw the line corresponding to cell number 2, and so on.

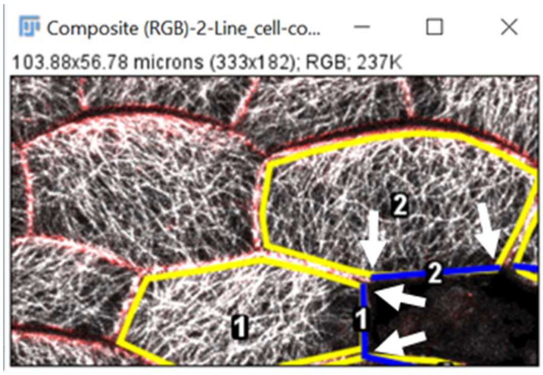

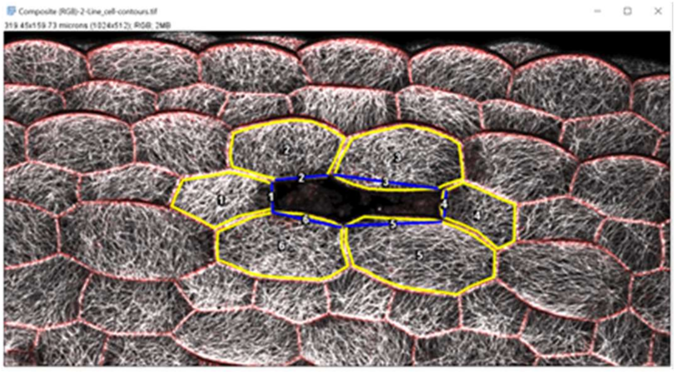

d. Check that each Line ROI number fits to the Cell ROI number, then click "OK" and proceed to the next sample.
