# Supplementary material for: High-throughput characterization of cortical microtubule arrays response to anisotropic tensile stress
Source: BMC Biol. 2023 Jul 10;21:154. doi: 10.1186/s12915-023-01654-7 (PMC10334548; doi:10.1186/s12915-023-01654-7)
Supplement: Supplementary file 3 — Additional file 3: Figure S1. High-throughput image analysis workflow to quantify CMT arrays in pavement cells. Figure S2. 3D vs. 2D context and rational for the analysis of projected signal from curved samples. Figure S3. Sample preparation and experimental design. Figure S4. Comparison of workflow output using manual estimation vs. geometry-based prediction of tensile stress pattern around the ablation. Figure S5. Plots of time series with different time resolutions. Figure S6. Time-lapse of the GFP-MBD reporter line after ablation or mock experiment. Figure S7. Individual sample plots of CMT arrays for the GFP-MBD reporter lines. Figure S8. Individual cell plots of CMT arrays for the GFP-MBD reporter lines. Figure S9. Time-lapse of the katanin mutant bot1-7 GFP-MBD reporter line after ablation or mock experiment. Figure S10. Individual sample plots of CMT arrays for the katanin mutant bot1-7 GFP-MBD reporter lines. Figure S11. Individual cell plots of CMT arrays for the katanin mutant bot1-7 GFP-MBD reporter lines. Figure S12. Time-lapse of the mCit-MBD reporter line after ablation or mock experiment. Figure S13. Individual sample plots of CMT arrays for the mCit-MBD reporter lines. Figure S14. Individual cell plots of CMT arrays for the mCit-MBD reporter lines. Figure S15. Time-lapse of the GFP-TUA6 reporter line after ablation or mock experiment. Figure S16. Individual sample plots of CMT arrays for the GFP-TUA6 reporter lines. Figure S17. Individual cell plots of CMT arrays for the GFP-TUA6 reporter lines. [file 12915_2023_1654_MOESM3_ESM.pdf]

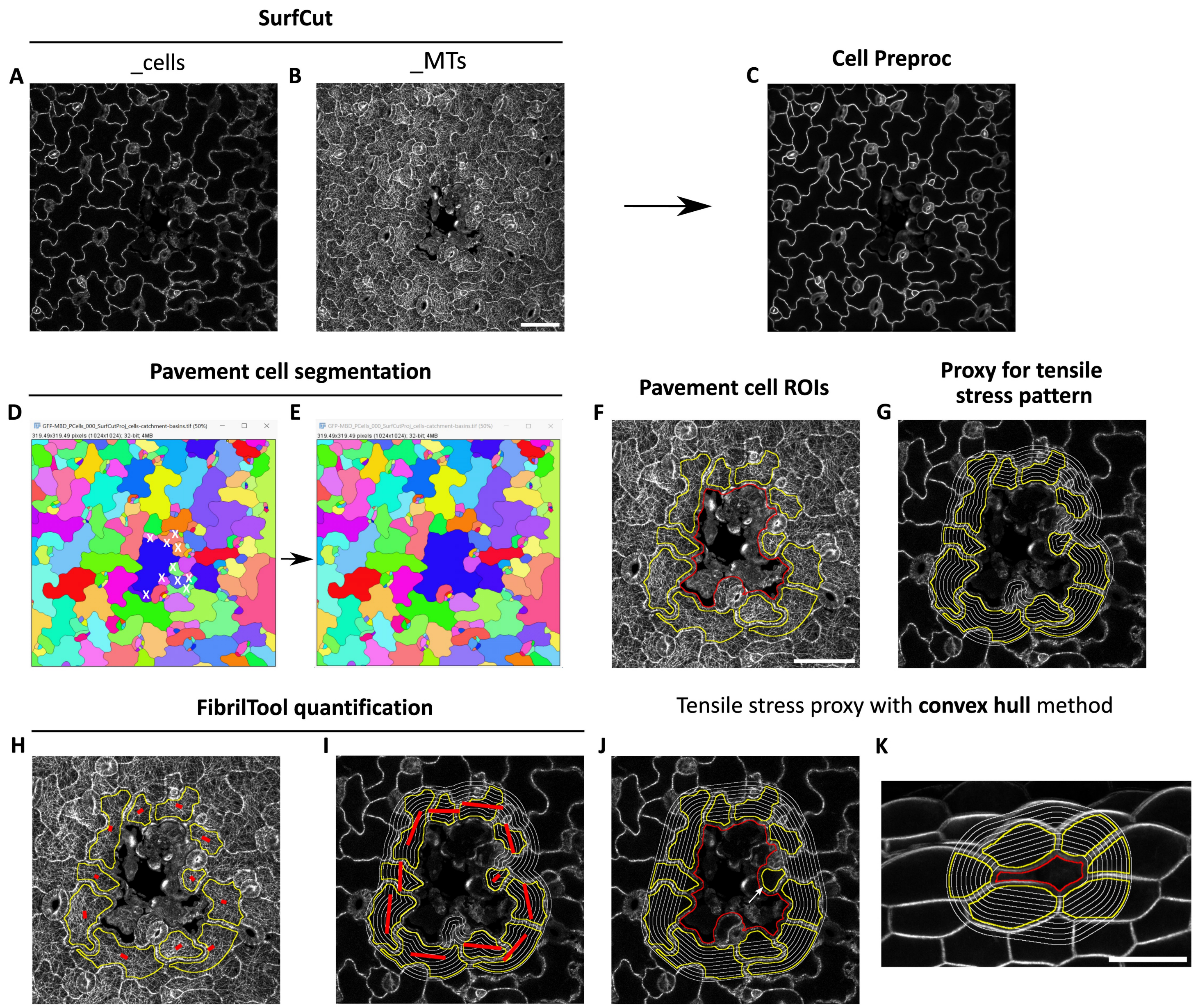

Supp. Figure S1. High-throughput image analysis workflow to quantify CMT arrays in pavement cells. Panels describe each step of the image analysis strategy in the case of an *Arabidopsis thaliana* cotyledon expressing the GFP-MBD microtubule reporter line. **(A,B)** Epidermal cell contours **(A)** and CMTs outer epidermal signal **(B)** extracted from the raw z-stack and projected in 2D using the SurfCut ImageJ macro. **(C)** Average projection image of several cell contour images aligned from the same sample timelapse using the "Cell Preproc" tool. **(D-G)** Description of the analysis process of the "ROI Maker" macro. **(D-E)** MorphoLibJ morphological segmentation of the cell contour image. Segmentation with errors highlighted by white crosses **(D)** and after manual correction **(E)**. The segmentation is successful on pavement cells despite small errors which can be corrected manually in the following steps of the macro either by merging some over-segmented areas or by manually designing an ROI using the polygon tool on imageJ. **(F)** 2D image of the outer epidermal CMTs overlayed with the ROIs of each cell regions surrounding the ablation (in yellow) and the ablation ROI (in red). **(G)** Image of the geometry-based proxy for tensile stress pattern, overlayed with the cell contour image and cell ROIs for context. **(H-I)** FibrilTool quantification of **(H)** the CMT arrays signal and **(I)** the tensile stress proxy in pavement cells. **(J,K)** Image of the geometry-based proxy representing the tensile stress pattern using the convex hull transformation of the ablation shape, overlayed with the cell contour image and cell ROIs for context on cotyledon **(J)** and hypocotyl **(K**; Same as **Figure 1** for comparison**)**. Users need to be aware when using the convex hull tool that some cells might be excluded from the analysis as shown in the case of pavement cells **(J)** this is not necessarily the case in the hypocotyl as shown in **K** and in **Figure 1J**. Scale bars are 50µm.

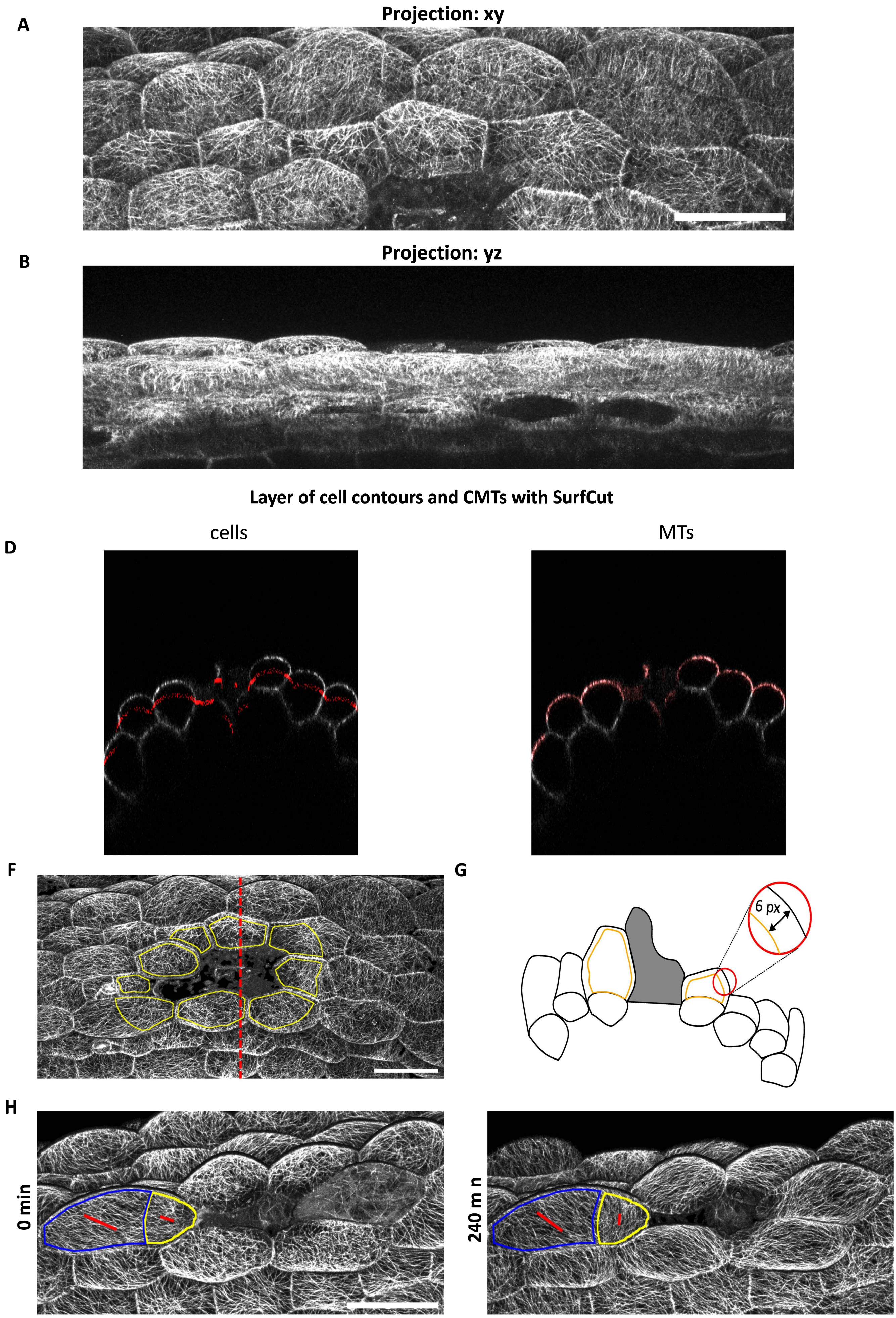

Supp. Figure S2. 3D vs. 2D context and rational for the analysis of projected signal from curved samples.

(A,B) Projection in xy **(A)** and yz **(B)** of a 3D confocal stack of a light grown hypocotyl. The distortion observed in the yz projection **(B)** is the consequence of the intrinsic anisotropy of 3D signal acquisition with a confocal microscope (point spread function). **(D,E)** Orthogonal views obtained with SurfCut, the red dots represent the signal extracted by SurfCut for cell contours **(D)** and cortical microtubules **(E)**. **(F)** 2D image of the outer epidermal CMTs overlayed with the ROIs of each cell regions to analyze surrounding the ablation (in yellow), the red dashed line represents the position of the orthogonal views in **D** and **E**. **(G)** Perspective drawing representing a section through a hypocotyl, cut through the middle of the ablation site. We represent the areas of analysis (ROIs, yellow) designed by our workflow and how the script segments the cell contour, then erodes 6 pixels from the cell contour to analyze the microtubules at the surface of cells and avoid bias of the higher signal of CMTs at the cell contours and curved sample in the analysis. In turn, the majority of the CMT signal quantified in our workflow is in a plan parallel to the 2D projection and should generally not be affected by projection or confocal signal anisotropy distortion. **(H,I)** FibrilTool quantification of the CMT arrays signal in "longer cells" normally trimmed 30µm from the centeredge of the ablation (yellow) and the remaining of the cell (blue) at times 0 **(H)** and 240min **(I)** after ablation. Both parts of the same cell react differently. When "close" to the ablation, potentially within the field of the new circumferential tensile stress, CMTs reorient whereas they don't appear to rearrange at a higher distance from the ablation in most cases. **H** and **I** display the same sample as the one used for illustration in **Figure1**. (Scale bars: 50µm)

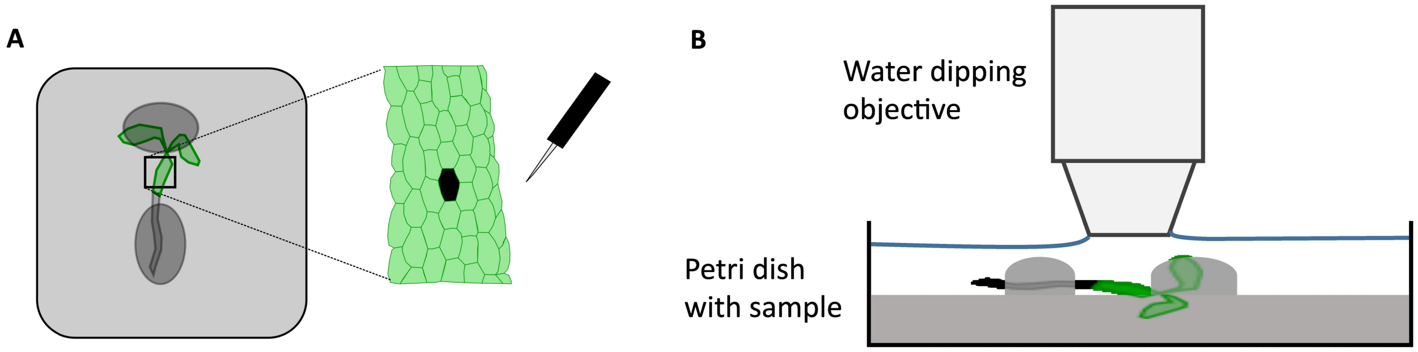

Supp. Figure S3. Sample preparation and experimental design. (A) Ablation of one or a few cells on the upper middle part of the hypocotyl immobilized at the cotyledons and the root on the plate containing the growth medium with low melting agarose (see material and methods). The ablation is performed with a fine needle minutiens on the upper middle part of the hypocotyl. **(B)** The plate with the sample is positioned under the water dip objective of an upright confocal microscope for time series imaging (see material and methods).

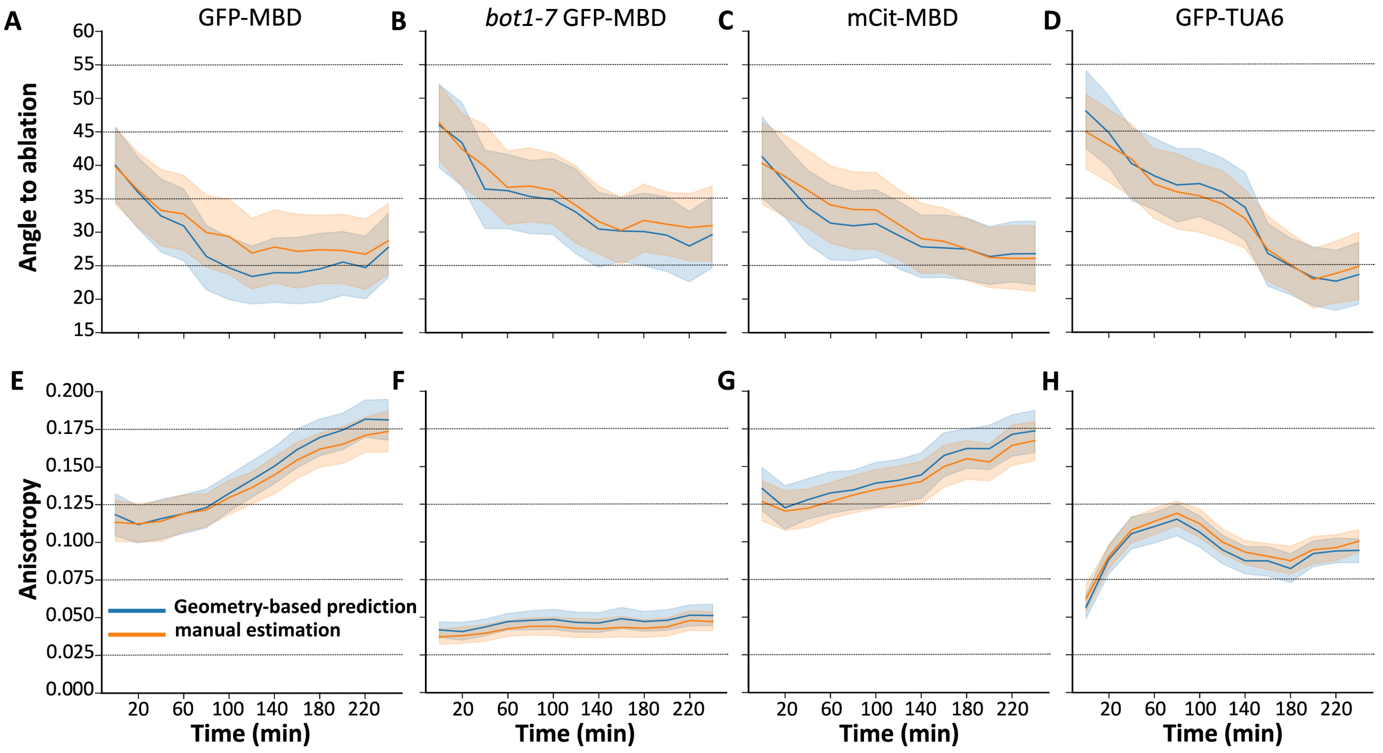

Supp. Figure S4. Comparison of workflow output using manual estimation vs. geometry-based prediction of tensile stress pattern around the ablation. Plots of the mean and 95% confidence interval for **(A-D)** angle to ablation and **(E-H)** anisotropy for **(A,E)** GFP-MBD, **(B,F)** bot1-7/GFP-MBD, **(C,G)** mCit-MBD and **(D,H)** GFP-TUA6. Data for the "geometry-based prediction" (blue lines) are the same as those displayed in **Figure 2** of the main text. In previous work, tensile stress orientation around the ablation was estimated by drawing lines along the cell faces adjacent to the ablation. Here, as a validation step, we have also performed the whole analysis using manually drawn references for tensile stress patterns for comparison (orange lines). While this approach is highly user biased, it can also be considered to bring user "expertise" into the analysis. Ultimately, we see no significant difference in the output of the two approaches which suggest that our method can be validated by "user expertise", while it removes further user bias.

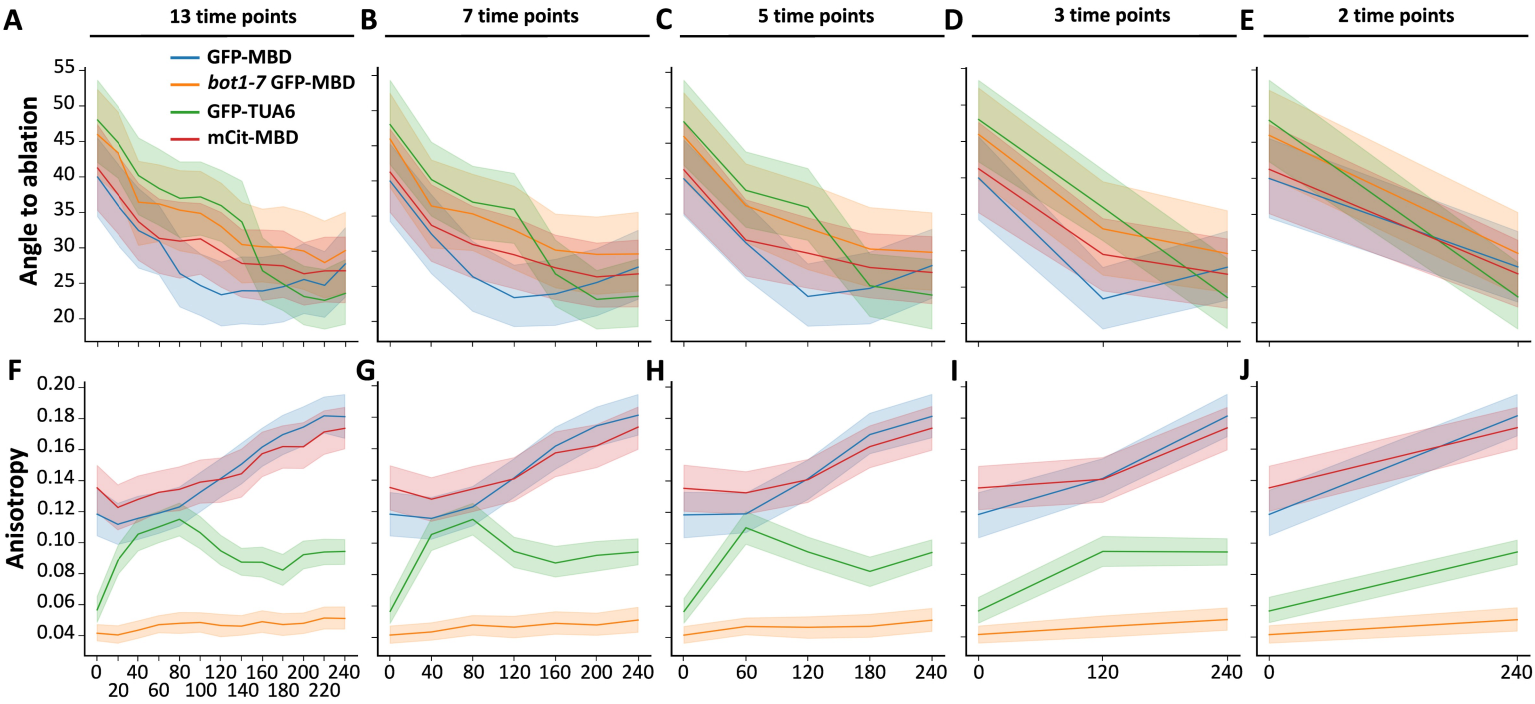

Supp. Figure S5. Plots of time series with different time resolutions. Plots from the same time series from 13 to 2 time points to illustrate the temporal resolution of CMTs reorganization over time. **(A-J)** Plots of the mean and 95% confidence interval for the angle to ablation **(A-E)** and anisotropy **(F-J)** comprising either 13 **(A, F)**, 7 **(B, G)**, 5 **(C, H)**, 3 **(D, I)** or 2 time points **(E, J)** in each investigated genotype. Graphs were generated by simply subsampling the original dataset **(A,F)**. Evidently, with less time points we lose information on the reorientation of CMTs over time, especially important for the GFP-TUA6 CMTs reporter line showing a strong response in the first hour after the tensile stress **(A, F)**.

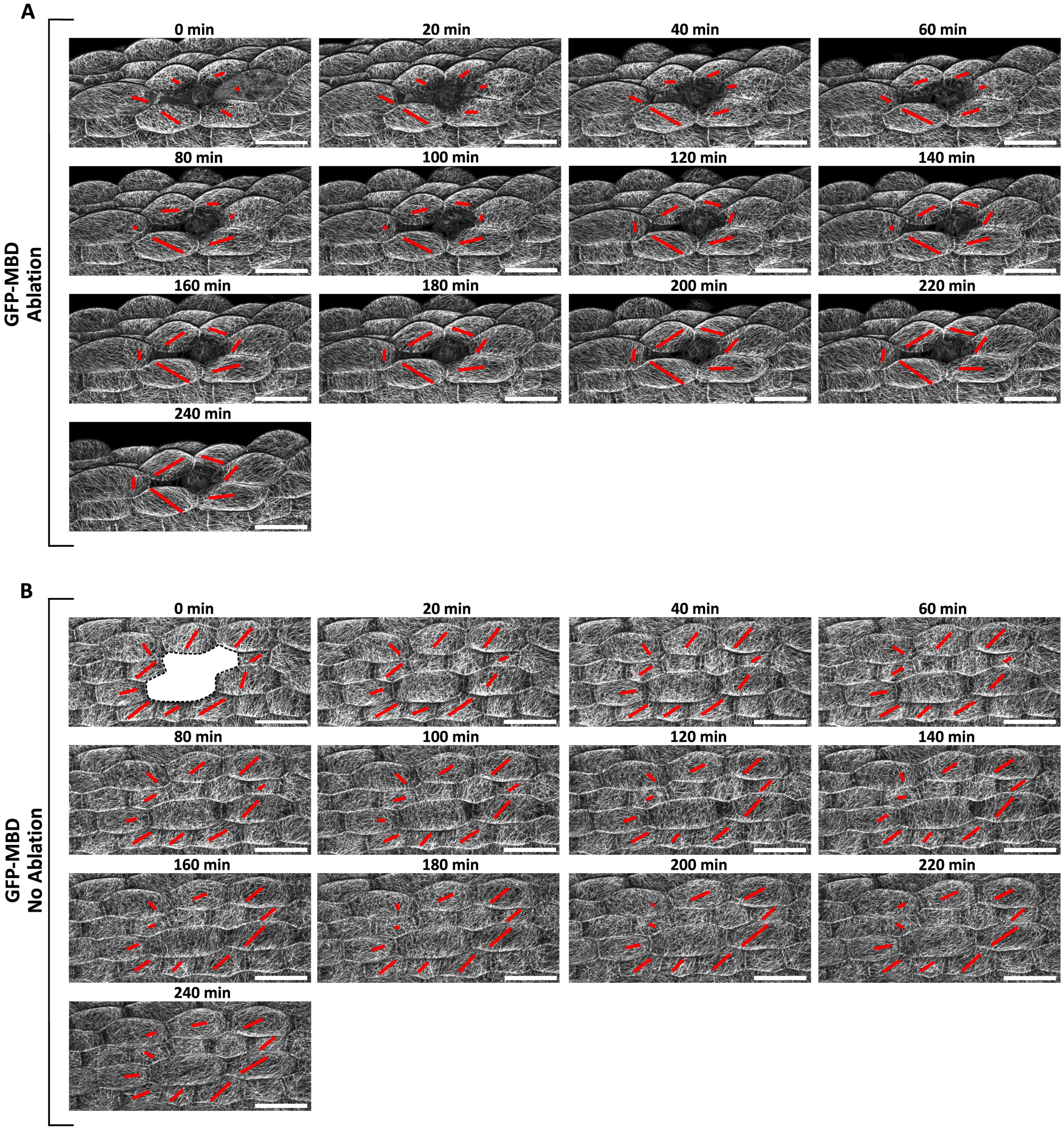

Supp. Figure S6. Time-lapse of the GFP-MBD reporter line after ablation or mock experiment. 2D projections of outer epidermal CMT signal (gray scale) overlayed with the visual output from FibrilTool quantification (red lines), from representative samples **(A)** with and **(B)** without ablation. The red lines from FibrilTool represent the main orientation of CMT arrays and their length represent the relative anisotropy of CMTs. Here, the FibrilTool line length representing the anisotropy has been multiplied by 2 and the width by 10 to the basic settings for better visual representation. The white area in **(B)** delimits the mock ablation chosen during the image analysis process. Scale bars are 50µm.

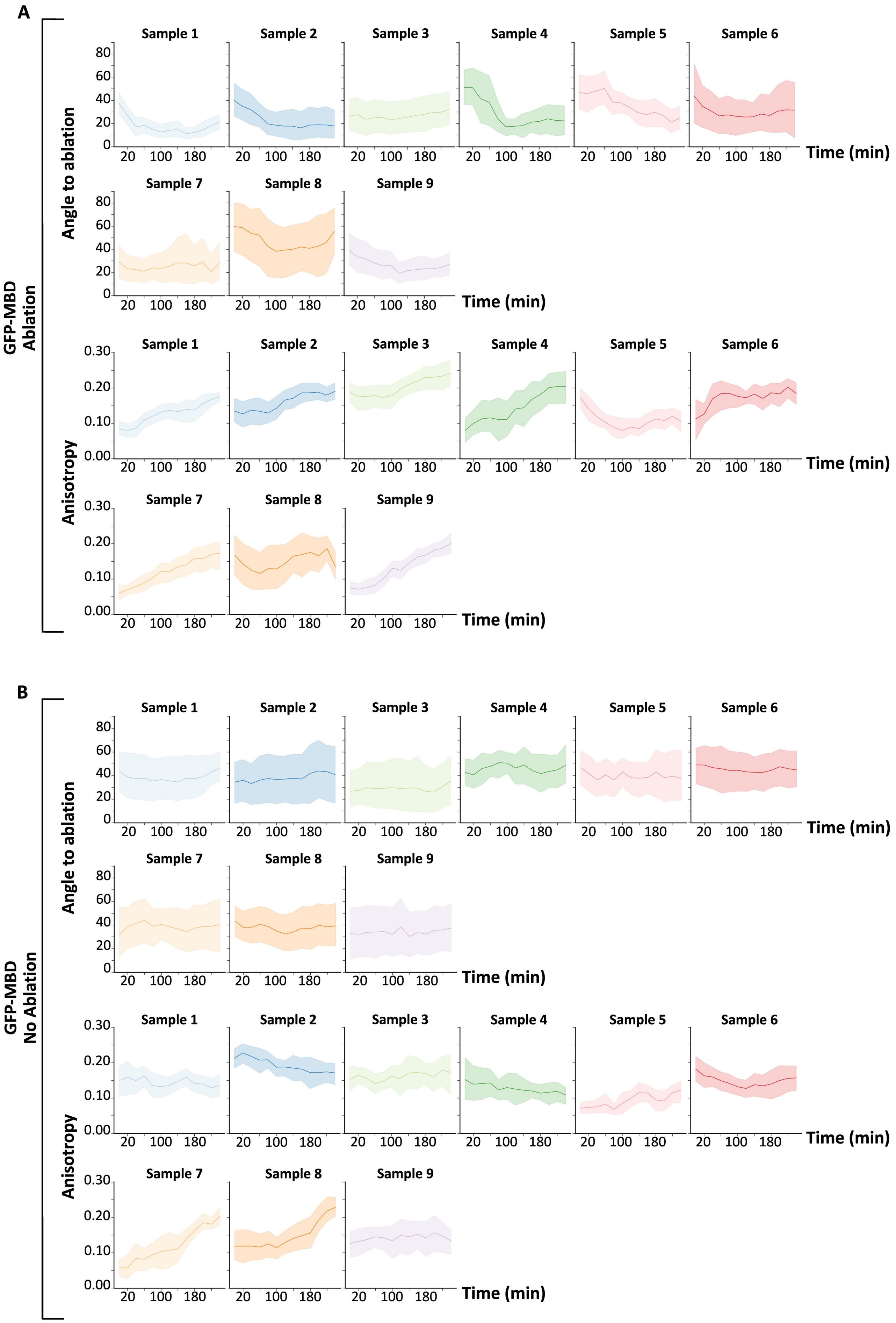

Supp. Figure S7. Individual sample plots of CMT arrays for the GFP-MBD reporter lines. Plots of the mean and 95% confidence interval for the angle to ablation and anisotropy values of each sample during the time series of 4 hours with a 20 minute interval after an ablation **(A)** and no ablation **(B)**.

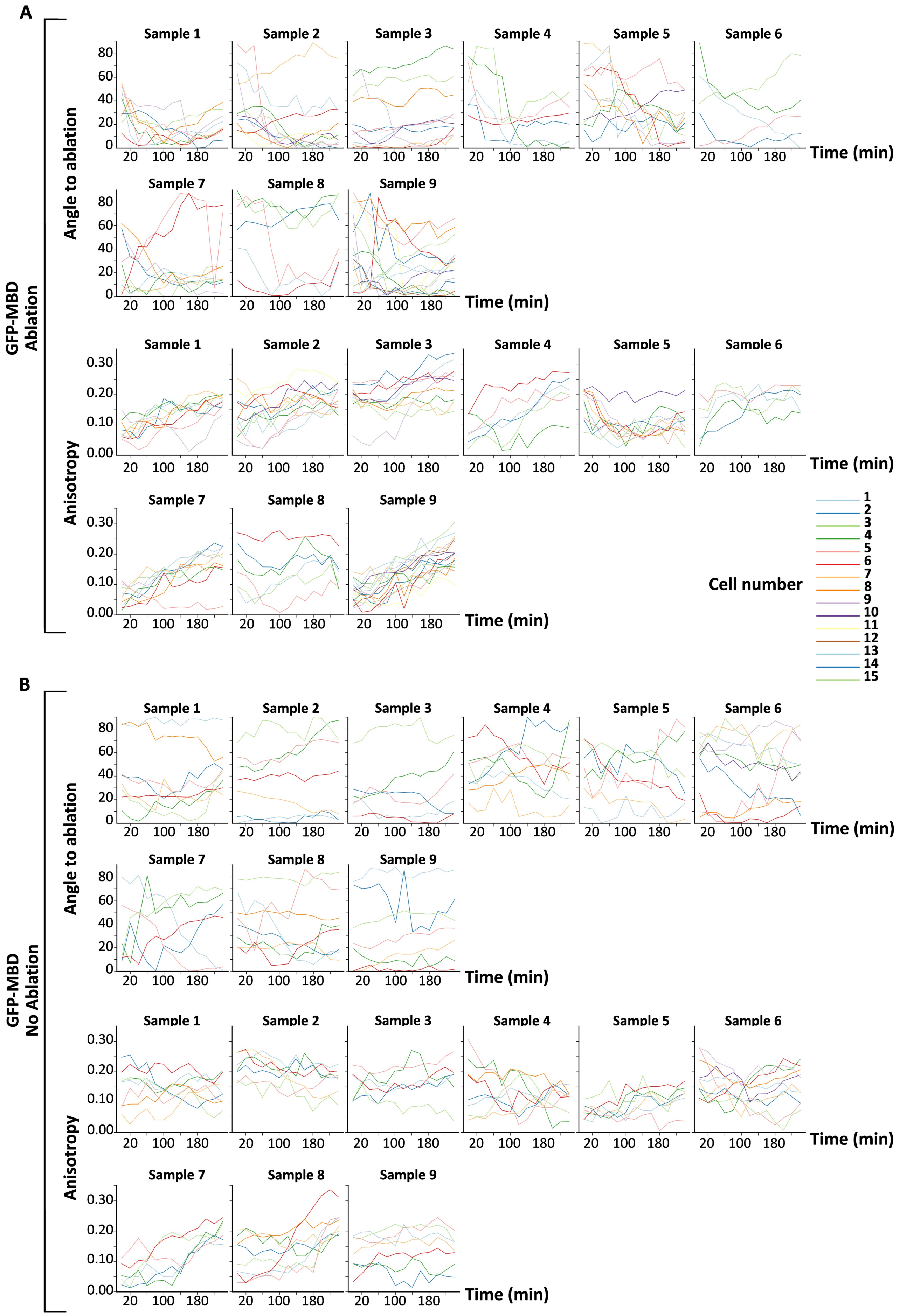

Supp. Figure S8. Individual cell plots of CMT arrays for the GFP-MBD reporter lines. Plots of the angle to ablation and anisotropy values in each individual cell of each sample during the time series of 4 hours with a 20 minute interval after an ablation **(A)** and no ablation **(B)**.

A

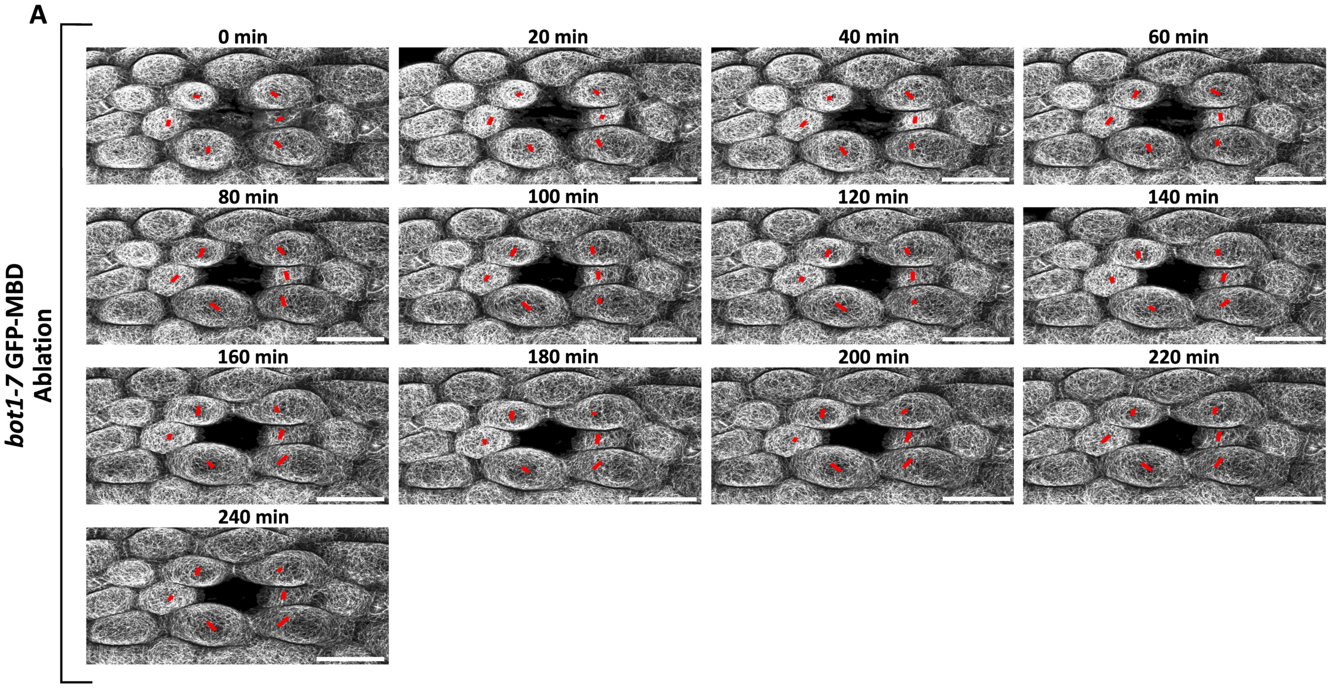

B

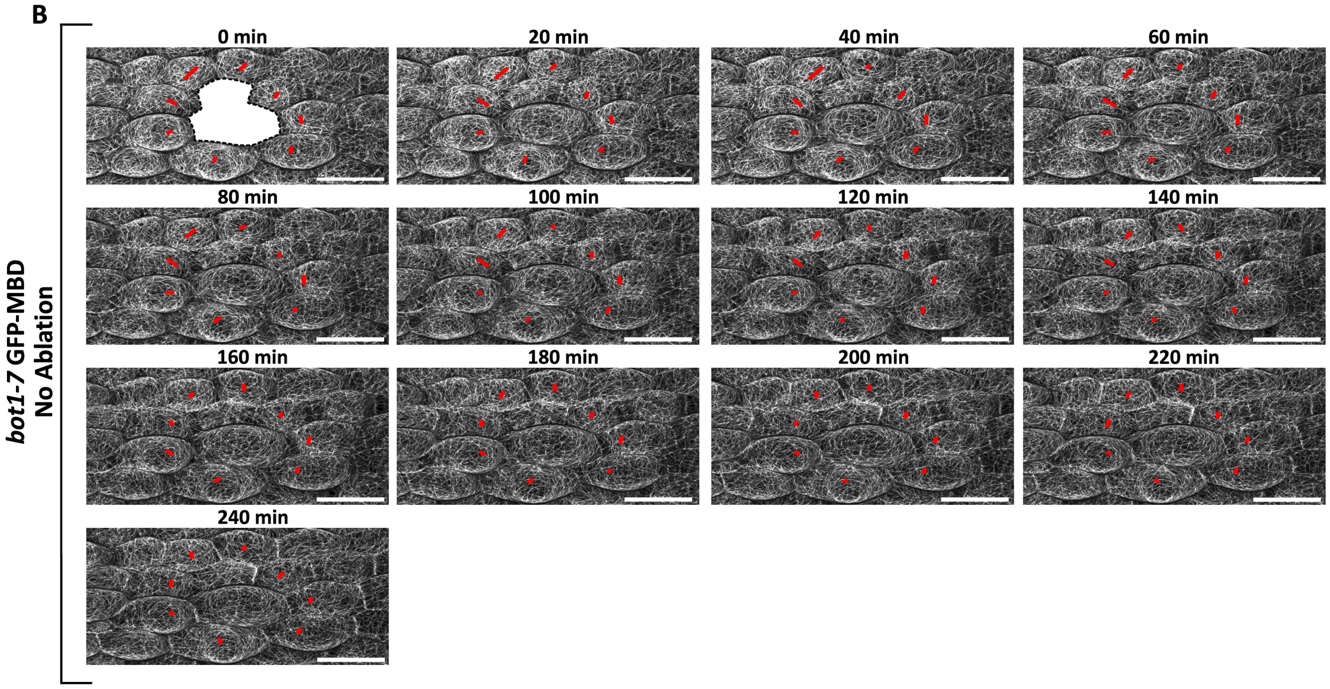

Supp. Figure S9. Time-lapse of the katanin mutant *bot1-7* GFP-MBD reporter line after ablation or mock experiment. 2D projections of outer epidermal CMT signal (gray scale) overlayed with the visual output from FibrilTool quantification (red lines), from representative samples **(A)** with and **(B)** without ablation. The red lines from FibrilTool represent the main orientation of CMT arrays and their length represent the relative anisotropy of CMTs. Here, the FibrilTool line length has been multiplied by 2 compared and the width by 10 to the basic settings for better visual representation. The white area in **(B)** delimits the mock ablation chosen during the image analysis process. Scale bars are 50µm.

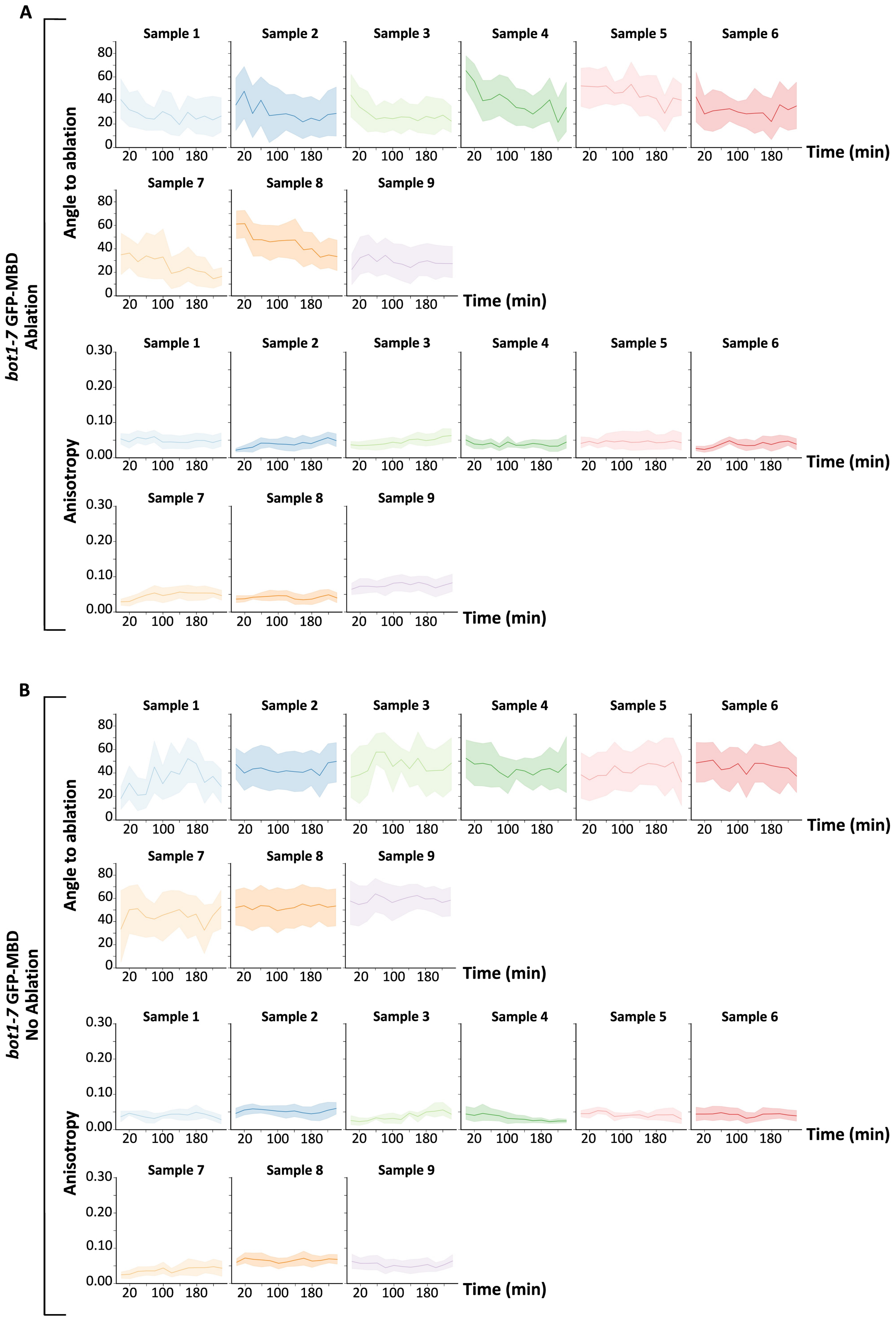

Supp. Figure S10. Individual sample plots of CMT arrays for the katanin mutant *bot1-7* GFP-MBD reporter lines. Plots of the mean and 95% confidence interval for the angle to ablation and anisotropy values of each sample during the time series of 4 hours with a 20 minute interval after an ablation **(A)** and no ablation **(B)**.

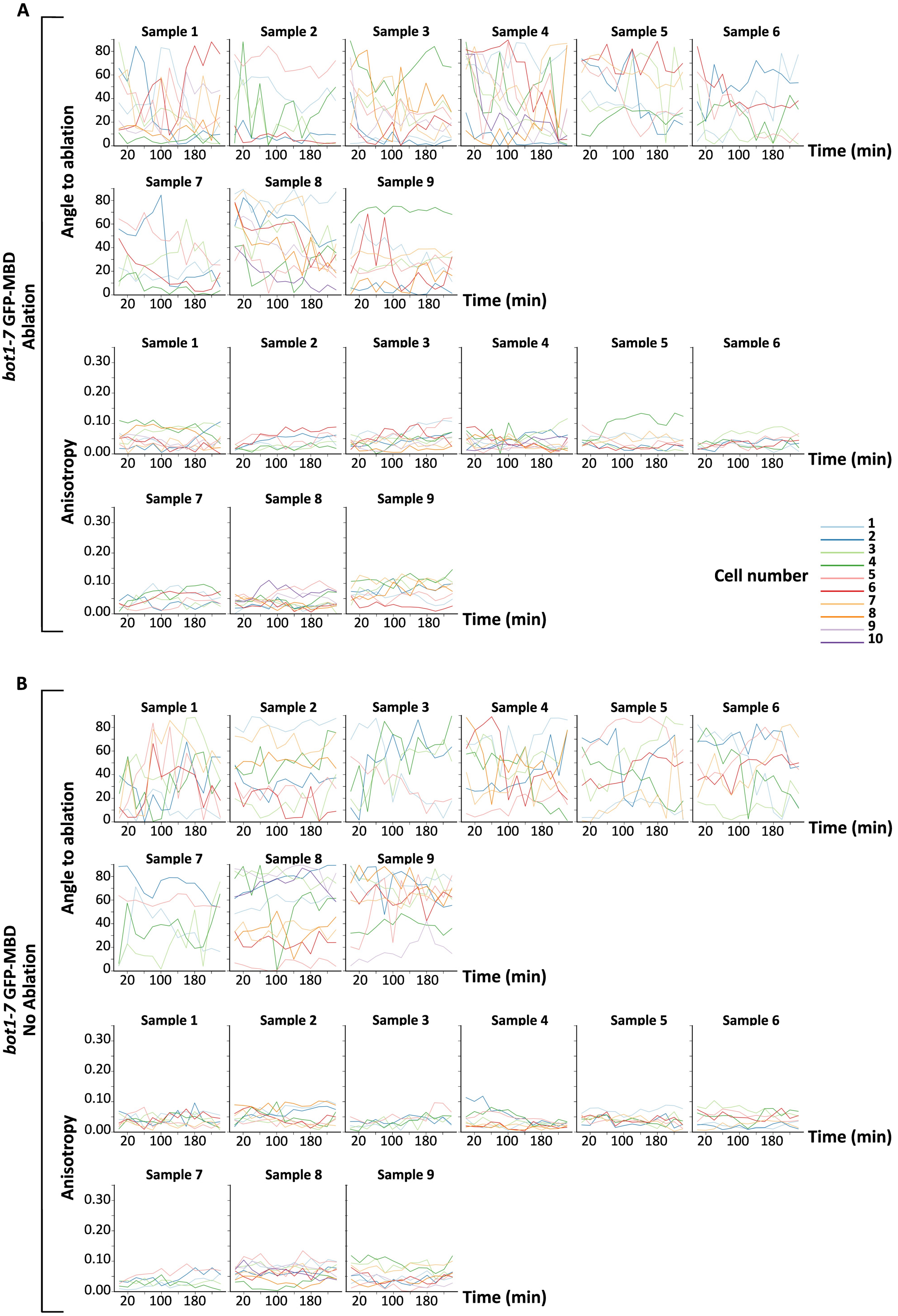

Supp. Figure S11. Individual cell plots of CMT arrays for the katanin mutant *bot1-7* GFP-MBD reporter lines. Plots of the angle to ablation and anisotropy values in each individual cell of each sample during the time series of 4 hours with a 20 minute interval after an ablation **(A)** and no ablation **(B)**.

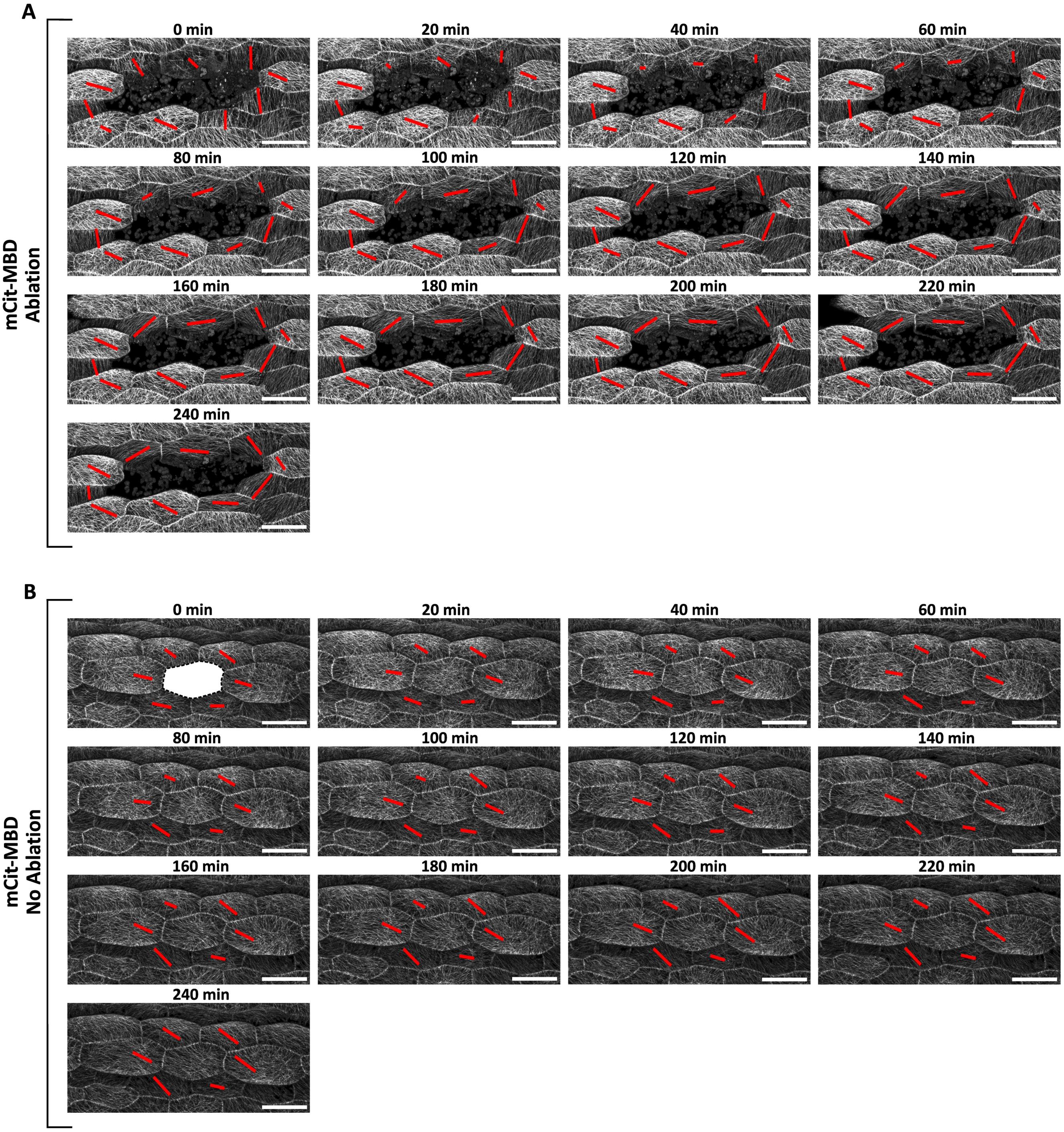

Supp. Figure S12. Time-lapse of the mCit-MBD reporter line after ablation or mock experiment. 2D projections of outer epidermal CMT signal (gray scale) overlayed with the visual output from FibrilTool quantification (red lines), from representative samples **(A)** with and **(B)** without ablation. The red lines from FibrilTool represent the main orientation of CMT arrays and their length represent the relative anisotropy of CMTs. Here, the FibrilTool line length has been multiplied by 2 compared and the width by 10 to the basic settings for better visual representation. The white area in **(B)** delimits the mock ablation chosen during the image analysis process. Scale bars are 50µm.

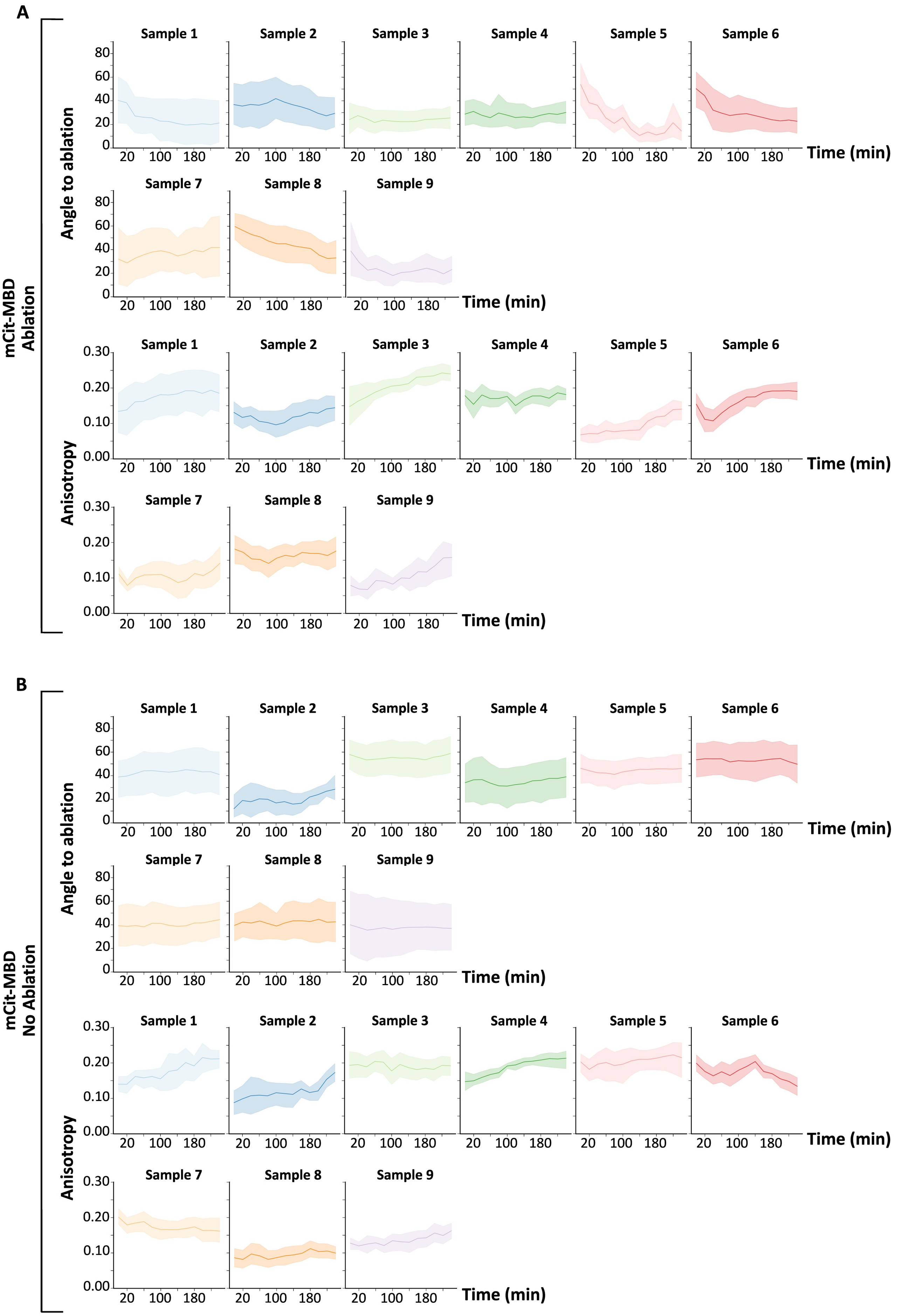

Supp. Figure S13. Individual sample plots of CMT arrays for the mCit-MBD reporter lines. Plots of the mean and 95% confidence interval for the angle to ablation and anisotropy values of each sample during the time series of 4 hours with a 20 minute interval after an ablation **(A)** and no ablation **(B)**.

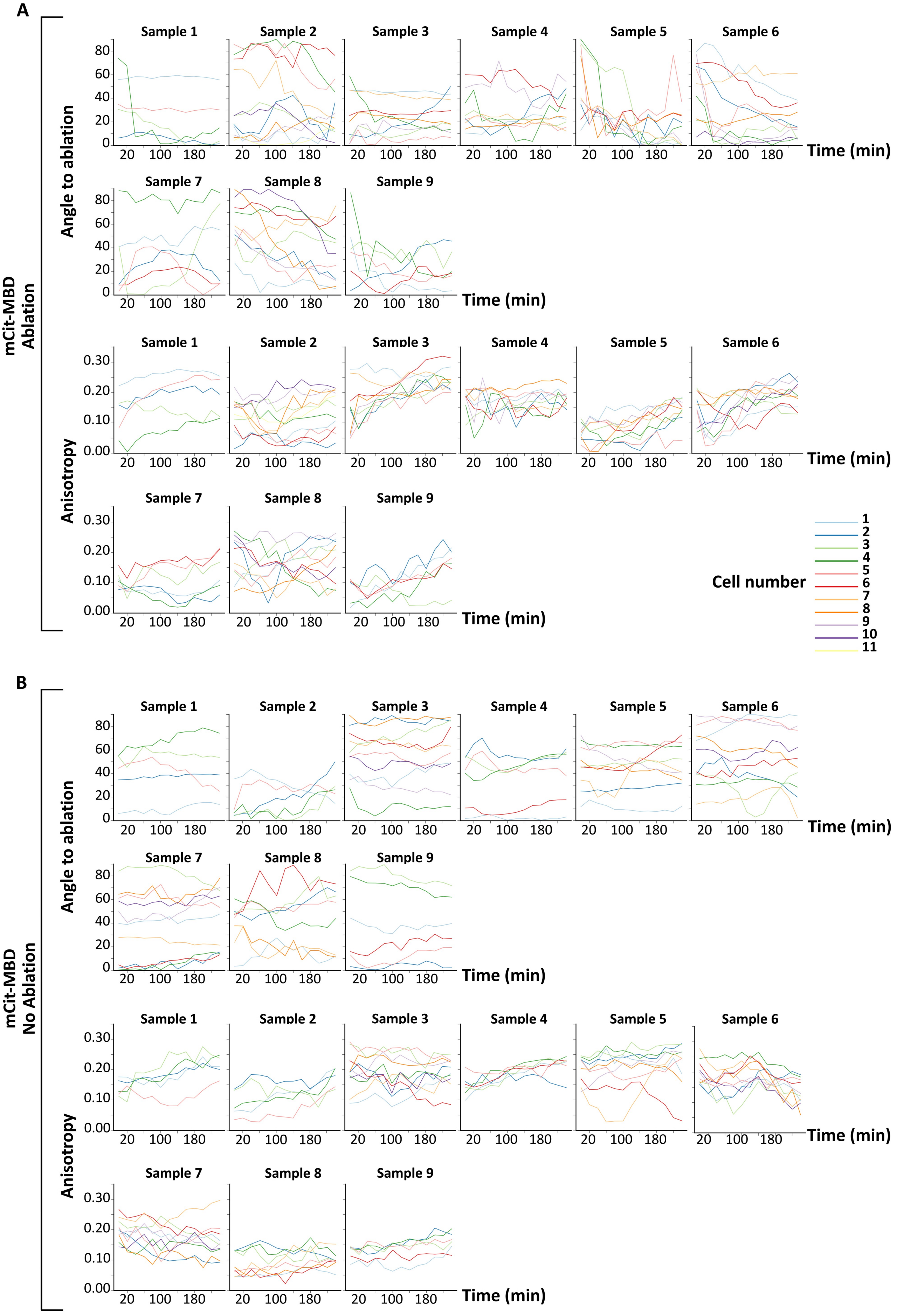

Supp. Figure S14. Individual cell plots of CMT arrays for the mCit-MBD reporter lines. Plots of the angle to ablation and anisotropy values in each individual cell of each sample during the time series of 4 hours with a 20 minute interval after an ablation **(A)** and no ablation **(B)**.

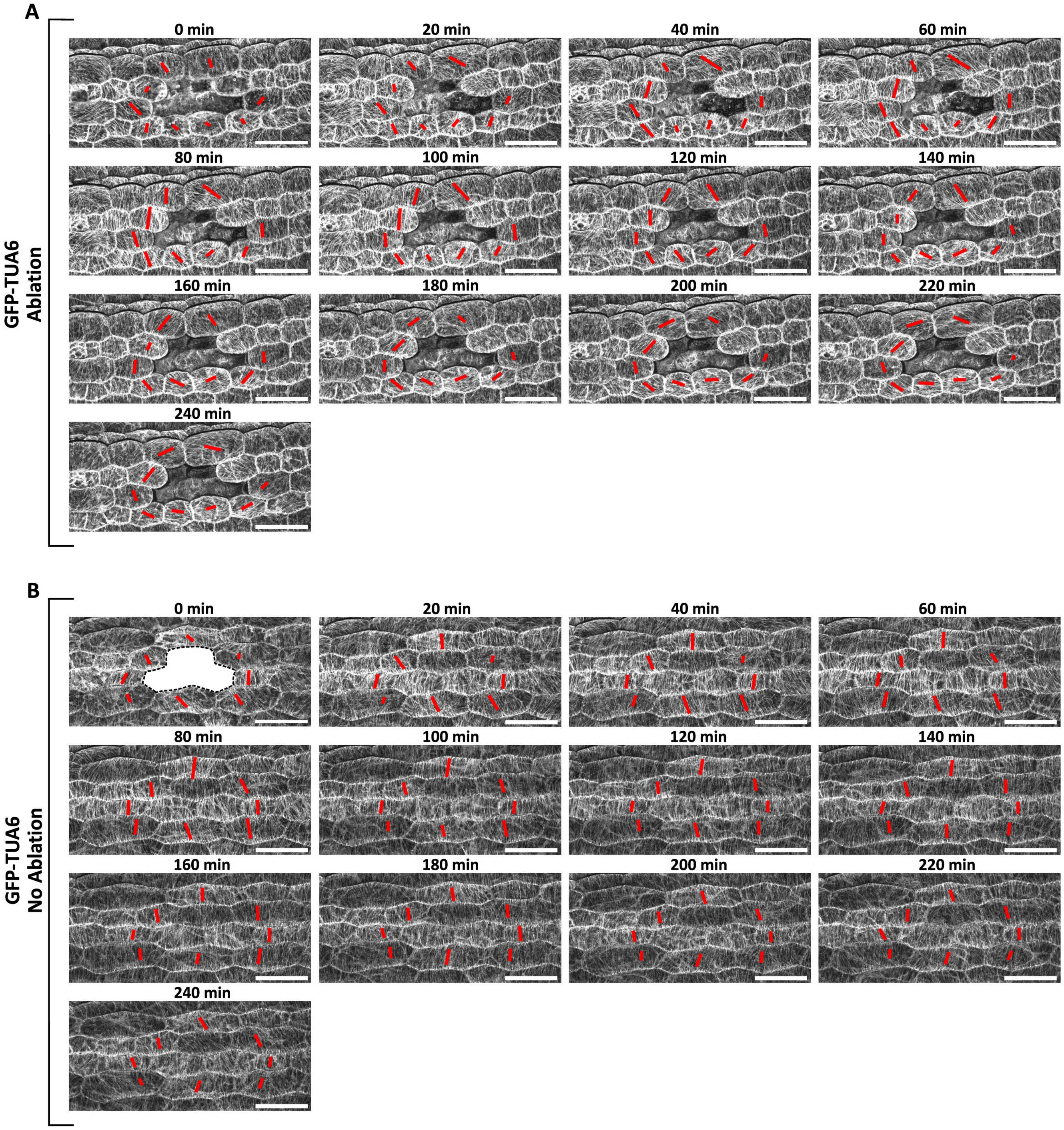

Supp. Figure S15. Time-lapse of the GFP-TUA6 reporter line after ablation or mock experiment. 2D projections of outer epidermal CMT signal (gray scale) overlayed with the visual output from FibrilTool quantification (red lines), from representative samples **(A)** with and **(B)** without ablation. The red lines from FibrilTool represent the main orientation of CMT arrays and their length represent the relative anisotropy of CMTs. Here, the FibrilTool line length has been multiplied by 2 compared and the width by 10 to the basic settings for better visual representation. The white area in **(B)** delimits the mock ablation chosen during the image analysis process. Scale bars are 50µm.

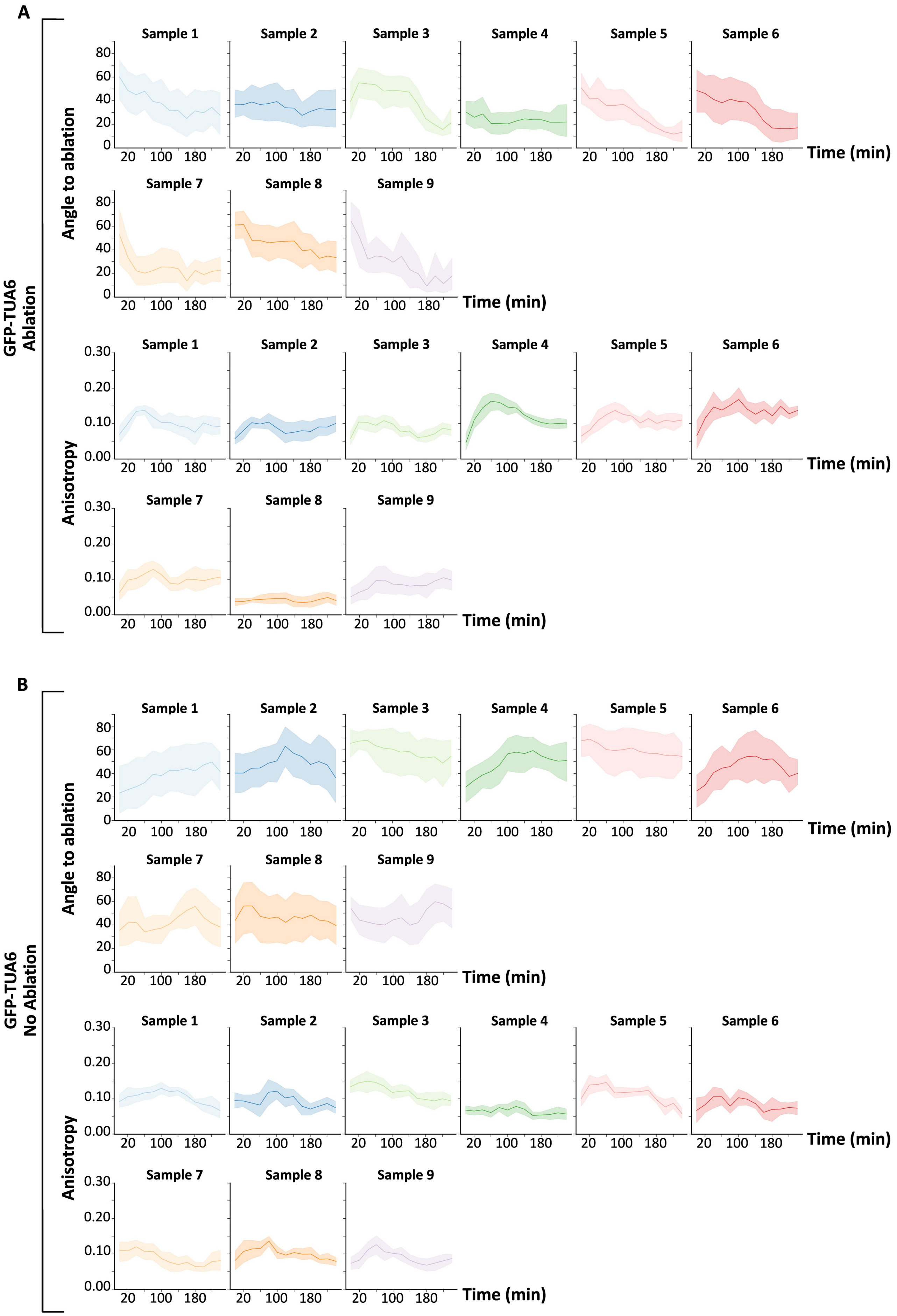

Supp. Figure S16. Individual sample plots of CMT arrays for the GFP-TUA6 reporter lines. Plots of the mean and 95% confidence interval for the angle to ablation and anisotropy values of each sample during the time series of 4 hours with a 20 minute interval after an ablation **(A)** and no ablation **(B)**.

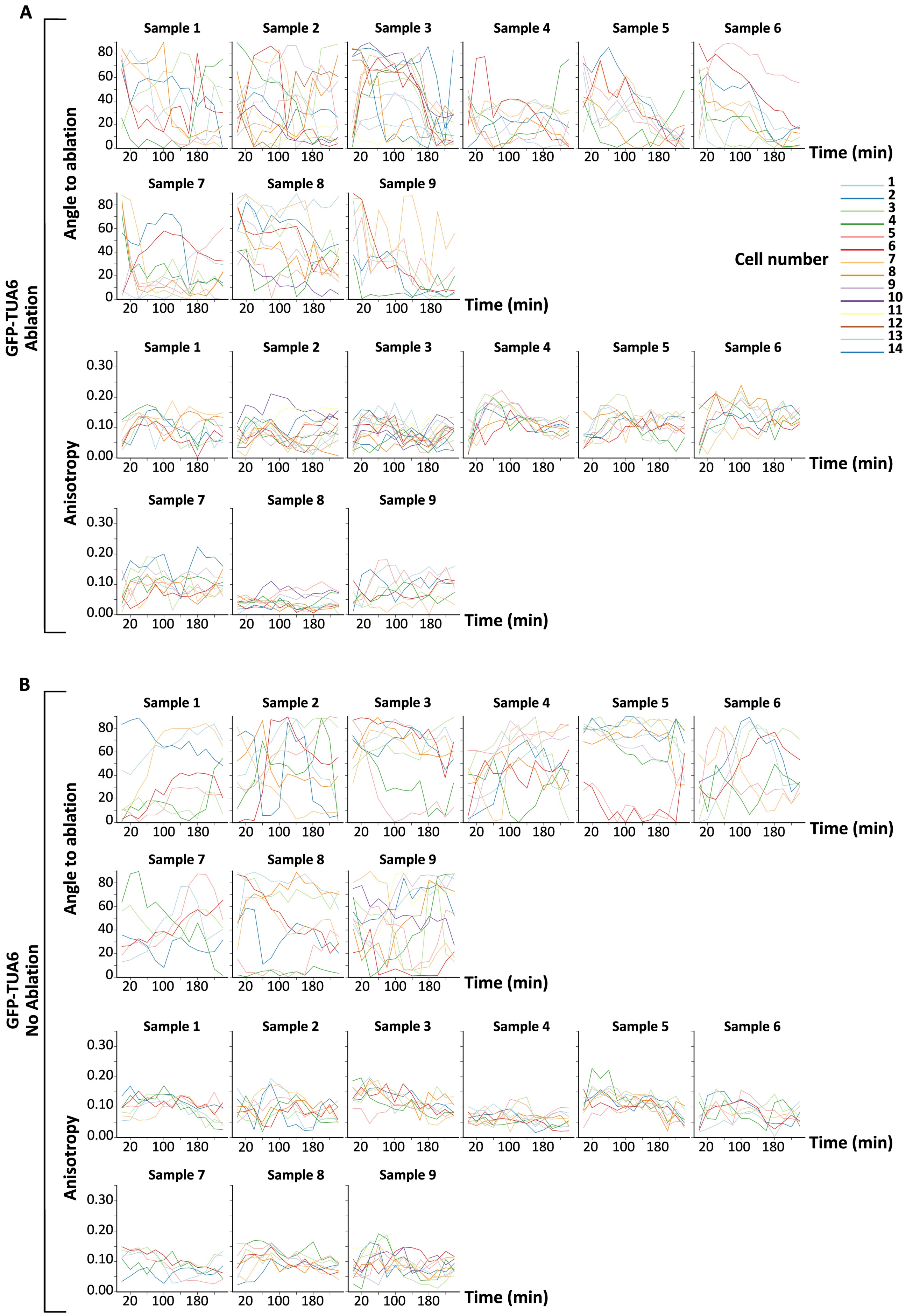

Supp. Figure S17. Individual cell plots of CMT arrays for the GFP-TUA6 reporter lines. Plots of the angle to ablation and anisotropy values in each individual cell of each sample during the time series of 4 hours with a 20 minute interval after an ablation **(A)** and no ablation **(B)**.
